# Genome-wide prediction models that incorporate *de novo* GWAS are a powerful new tool for tropical rice improvement

**DOI:** 10.1038/hdy.2015.113

**Published:** 2016-02-10

**Authors:** J E Spindel, H Begum, D Akdemir, B Collard, E Redoña, J-L Jannink, S McCouch

**Affiliations:** 1 Department of Plant Breeding and Genetics, 240 Emerson Hall, Cornell University, Ithaca, NY, USA; 2 Department of Plant Breeding, Genetics and Biotechnology, International Rice Research Institute, Los Baños, Philippines; 3 USDA-ARS, North Atlantic Ares, Robert W. Holley Center for Agriculture and Health, Ithaca, NY, USA

## Abstract

To address the multiple challenges to food security posed by global climate change, population growth and rising incomes, plant breeders are developing new crop varieties that can enhance both agricultural productivity and environmental sustainability. Current breeding practices, however, are unable to keep pace with demand. Genomic selection (GS) is a new technique that helps accelerate the rate of genetic gain in breeding by using whole-genome data to predict the breeding value of offspring. Here, we describe a new GS model that combines RR-BLUP with markers fit as fixed effects selected from the results of a genome-wide-association study (GWAS) on the RR-BLUP training data. We term this model GS + *de novo* GWAS. In a breeding population of tropical rice, GS + *de novo* GWAS outperformed six other models for a variety of traits and in multiple environments. On the basis of these results, we propose an extended, two-part breeding design that can be used to efficiently integrate novel variation into elite breeding populations, thus expanding genetic diversity and enhancing the potential for sustainable productivity gains.

## Introduction

Rice (*Oryza sativa*) is a model species for crop genomics. In 2002, rice became the first crop species to have its genome sequenced (Goff *et al.*, 2002), and it remains one of the best characterized crop genomes due to its small size, abundant genetic variation and high-quality sequence data (Ronald and Leung, 2002; Huang *et al.*, 2013). Since the sequencing and annotation of the rice genome, there have been >4278 publications on rice genetics, ~3000 genes have been structurally and/or functionally annotated (Ouyang *et al.*, 2007) and 3000 rice genomes have recently been sequenced at ~10X coverage (Alexandrov *et al.*, 2014). Although these numbers represent great progress for rice biology, they have so far had little impact on rice agriculture (Bernardo, 2008).

One of the major challenges facing the agricultural community as it seeks to integrate genomic information into applied plant improvement has been that until recently genotyping was expensive and laborious (Desta and Ortiz, 2014; Varshney *et al.*, 2014; Lipka *et al.*, 2015). As a result, genomic applications were constrained by the number of marker data points that could be assayed per generation in large breeding populations. Consequently, most of the characterized genes in rice are those associated with Mendelian traits, that is, traits controlled by a few genes of large effect. The majority of agronomic traits of interest to plant breeders, however, are quantitative and polygenic, governed by many genes of small effect.

This paradox has determined how molecular markers and genomic information have been applied to breeding programs. In particular, it has lent itself to marker-assisted selection (MAS) in rice as well as in a variety of other species. In all cases, markers in critical large effect genes or genomic regions are used to predict the presence or absence of agriculturally valuable traits (Collard *et al.*, 2005; Bernardo, 2008; Collard and Mackill, 2008). Although MAS contributed to shortening the time required to develop and release new rice varieties, from ~12 years to ~6 years, it is predicated on prior knowledge about major-effect genes and quantitative trait loci (QTLs) that serve as the targets of selection. Furthermore, MAS is generally used to introduce one gene at a time into an existing and highly valued variety. It is not designed to manage the recombination of many genes simultaneously (Collard and Mackill, 2008; Desta and Ortiz, 2014).

Genomic Selection (GS) offers an alternative to MAS and conventional phenotypic selection. GS has the capacity to use full-genome data to increase breeding efficiency. In GS, a training population is phenotyped for a trait(s) of interest and genotyped using genome-wide markers. A statistical/machine learning model is built from the training population data that take the genotypes of individuals in a breeding population as input and outputs a measure of the value those individuals hold as parents of future breeding – the genome estimated breeding value (GEBV; Meuwissen *et al.*, 2001; Lorenz *et al.*, 2011). GS improves breeding efficiency by providing GEBVs for all individuals in a population, thus enabling the breeder to make informed decisions about which individuals to use in crossing or in allocating phenotyping resources among individuals and generations. Appropriate use of GEBVs saves time, effort and money in a breeding program by reducing the requirement to phenotype the entire population every generation, increasing the proportion of top performers in the breeding population, and enabling selection based on allele rather than line means (Heffner *et al.*, 2009; Bernardo, 2010). It can also accelerate the delivery of new varieties by keeping the breeding pipeline full of high-quality material. Unlike in MAS, in GS even infinitesimally small effect alleles contribute to model development, and are thus tracked and accounted for by the model. As a result, many loci can be under selection simultaneously. GS is known to be effective for maize and small grains, and recent studies suggest it will also be useful for rice (Asoro *et al.*, 2013; Massman *et al.*, 2013b; Crossa *et al.*, 2014; Onogi *et al.*, 2015; Spindel *et al.*, 2015).

Depending on the crop, trait, and breeding population design, the choice of statistical method used to build the GS model has been shown to have a significant effect on prediction accuracy (Daetwyler *et al.*, 2013). Interestingly, for breeding crops such as rice and wheat, in which large effect QTL are common, models that incorporate a select number of molecular markers as fixed effects have been shown to contribute to improved prediction accuracy. Bernardo (2014) first proposed based on simulation experiments that when 1–3 major genes for a trait are known and each accounts for ⩾10% of the phenotypic variance that these genes should be included as fixed effects in GS models (Bernardo, 2014). Empirical results by Rutkoski et al., 2014 confirmed the utility of the strategy for stem rust resistance in a wheat breeding population, while Owens *et al.* (2014) showed that it was effective for predicting pro-vitamin A content in a maize diversity panel. Other groups showed similar utility by weighting known genes of large effects in wheat (Bentley *et al.*, 2014; Zhao *et al.*, 2014). It is currently unknown, however, how including markers as fixed effects will improve rice GS models.

A second open question is how markers to be fit as fixed effects should be identified and/or selected. The previously cited experiments have, for the most part, relied on known functional markers for traits of interest (Bentley *et al.*, 2014; Owens *et al.*, 2014; Rutkoski *et al.*, 2014; Zhao *et al.*, 2014), or propose using previously published genome-wide association study (GWAS) results (Zhang *et al.*, 2014). Here, we propose for the first time directly using only the results of GWAS run using GS training population data, a method we are calling ‘GS + *de novo* GWAS', or simply ‘GS+GWAS'. There are numerous benefits to this approach. Both GS and GWAS use the same input data sets, a phenotype data set and a genotype data set, thus no additional data are required, only additional analysis. Furthermore, we hypothesize that the significant single-nucleotide polymorphisms (SNPs) identified from *de novo* GWAS will be more directly relevant to the population undergoing selection than SNPs identified as significant in previously published GWAS on potentially disparate populations, and thus will improve prediction accuracy beyond that which could be obtained using previously published GWAS results. Using *de novo* GWAS as part of the GS model is also more accessible to breeders, as it does not require an extensive knowledge or literature search on the underlying genetics of a trait of interest.

There are several points in a standard pedigree breeding program where an intervention based on GS could significantly shorten the breeding cycle by eliminating a generation of phenotyping or providing the breeder a mechanism for eliminating poor-performing offspring before the next generation of costly field trials (Spindel *et al.*, 2015). In this study, we assess the potential of introducing fixed variables identified using *de novo* GWAS into GS models to improve prediction accuracy as compared with GS models that make use of historical GWAS data or other standard GS models, and also consider the contribution of multi-location field trials to GS prediction accuracy. Using available multi-environment trial (MET) data from the International Rice Research Institute (IRRI) irrigated rice breeding program, we extract information from our models to evaluate which of currently used environments in IRRI's MET program can be combined for the purposes of model training, and thus define the best combination of environments for trait modeling and prediction in Southeast (SE) Asia.

The current study was undertaken as part of a rigorous evaluation of the irrigated rice breeding program at IRRI, and was specifically designed to investigate opportunities for integrating GS into that program. The data sets presented here were collected in eight locations in SE Asia between 2009 and 2012, and are used to highlight opportunities to use existing unbalanced phenotypic data sets in combination with genotyping-by-sequencing genotype data to develop and optimize GS models. Given that the cost of genotyping is continuing to decline while costs of field-based phenotyping are high and generally increasing, it is time for the rice breeding community to consider if and how GS can be implemented in rice breeding programs and to define the pilot breeding studies that can be used to help test and transition to genomics-assisted selection methods.

## Materials and methods

### Plant material and phenotyping

In all, 369 elite breeding lines (F_6_–F_7_) were selected for genotyping from the IRRI irrigated rice breeding program based on the planned inclusion of the lines in the 2011 Multi-Environment Testing Program and presence in the 2011 and 2012 Replicated Yield Trials (RYT) at IRRI (Los Baños).

Two phenotype data sets were used in this study, (1) the RYT data set, consisting of field data from 2009–2012, two seasons per year (dry season (DS) and wet season (WS)) collected in a single field at IRRI in Los Baños, Philippines, and (2) the MET data set, consisting of field data from 2011 and 2012, two season per year (dry and wet), at a total of eight sites in SE Asia (Table 1).

For the RYT data set, phenotypes collected included plant height, flowering time, maturity date, number of effective tillers or panicles per plant, lodging score, grain yield and rep number (Supplementary Materials and Methods).

For the MET data set, in addition to the phenotypes collected for the RYT data set, data were collected on field row, field column, phenotypic acceptability score for whole plant, phenotypic acceptability score for panicle and phenotypic acceptability score for grain (Supplementary Materials and Methods). The eight sites at which the MET data were collected to compose IRRI's target population of environments for irrigated rice in SE Asia including IRRI/Los Baños (‘MET field'), Isabela, Nueva Ecija, Agusan del Norte, Bohol, and Midsayap—all in the Philippines, Batalagoda, Sri Lanka, and Hai Dong, Vietnam. Data were highly unbalanced (Table 1; Supplementary Materials and Methods).

### Genotyping

Genotyping by sequencing was performed on 369 breeding lines as described in Spindel *et al.*, 2015 (Spindel *et al.*, 2015). For details on analysis and filtering see the Supplementary Methods. The cross-validation (CV) results reported in Supplementary Tables S2 and S4 were obtained using a genotype data set consisting of 108,005 SNPs with call rates ⩾0.75 and monomorphic SNPs removed. CV results reported in Supplementary Table S3 were obtained using subsets of this SNP data set after removing SNPs with minor allele frequency <0.05 (subsets were thus selected from a total of 58 318 SNPs, minor allele frequency filtering was performed for purposes of making GWAS more robust), for details on selection of SNP subsets, see 'CV using SNP subsets' below. For all data sets, after SNP filtering, individuals with >60% missing data were dropped from the data set, which resulted in the removal of six individuals that failed sequencing for a total of 363 genotyped lines. Heritabilities (Table 1) were calculated using a subset of the 108 005 SNPs with call rates ⩾90%.

### Subpopulation and family structure analysis and CV fold design

The majority of the 363 lines were characterized *a priori* from pedigree records as belonging to the *indica* or *indica-admixed* subpopulation groups. To identify outlier individuals belonging to the *japonica* or *japonica-admixed* groups, principal components analysis was performed in R (version 3.0.1; https://www.r-project.org) using a 73 147 SNP subset of the 108 005 SNPs used for GS with imputed call rates ⩾0.9 (remaining missing data were then filled using the line means). The results of the principal components analysis were used to identify 31 subpopulation outliers belonging to either the *japonica* subgroup or containing substantial *japonica* admixture. These 31 outliers were removed from the data set and were not included in any further analyses (Spindel *et al.*, 2015).

It was also known from studying the breeding program pedigrees that differing degrees of family relatedness existed within the remaining 332 lines, including half sibs, full sibs, parents and offspring, and unrelated lines. The presence of highly related individuals in the data set could have the effect of artificially inflating prediction accuracy if the most closely related individuals are randomly assigned to different folds, and one of those folds is then used as training, whereas the other is used as testing. To control for this possibility when designing our folds, we performed a partitioning around k-medoids analysis (pamk) using the R fpc package (function pamk; https://cran.r-project.org/web/packages/fpc/index.html) with the 73 147 imputed SNPs (Hennig, 2015; Kaufman and Rousseeuw, 1990). The largest average silhouette width was found to occur at *k*=87, so individuals found within the same cluster of 87 were assigned to the same fold, making it impossible for the most closely related individuals to be split across training and testing folds. Full clusters were assigned to one of five folds randomly, controlling only for cluster size to produce three folds of 66 individuals and two folds of 67 individuals. A similar procedure was used by Ly *et al.* (2013).

### CV experimental design

For each CV experiment, one of the five folds served as the validation fold, and the other four folds served as the training folds. The process was repeated five times so that each fold served once as the validation fold, resulting in predicted GEBV values for all individuals. Accuracy was assessed as the mean Pearson correlation of the predicted GEBV and adjusted phenotype in the validation population. For the RYT data, CV experiments were performed to test all logical combinations of years and seasons in the training and validation populations. A year's WS was never used to predict the same year's DS because in SE Asia, the DS arrives first chronologically. We did, however, predict the 2012 WS both with and without the preceding 2012 DS present in the training population. We tested scenarios in which both seasons per year were included in the training population, as well as scenarios where only the data from the seasons matching the validation population were included in the training data (for example, using only the WS data to predict the WS). We also sought to test scenarios using only more recent year data in the training population (for example, only 2011, or 2010–2011) and scenarios using more historical year data in the training population (for example, 2009–2011; Supplementary Table S1A).

The same logic was applied to the combination of years and seasons for the different MET CV experiments. In addition, the MET experiments were varied in terms of the site composition in the training population. In addition to CV experiments in which all sites were combined into a single training population and only the validation site was used to compose the training population, other combinations of MET sites were chosen based on (1) the geographic location of the sites, (that is, more northern sites were placed together, more southern sites were placed to together, where sites in between the northernmost and southernmost sites were tested in a variety of groupings) and (2) phenotypic data correlation, that is, if sites appeared to be correlated for any of the phenotypes of interest, they were tested in combination (Supplementary Table S1B). Note that Vietnam was excluded from all experiments except the 'validation site only' experiments because it was not correlated with any other site (Supplementary Figure S1). The 2012 Isabela DS was both included and excluded in any CV experiment in which it would normally have been included because it also was not correlated with any other site or season, including other Isabela years/seasons (Supplementary Figure S1). For additional details on MET experiments, calculation of adjusted phenotypes for validation folds and correlation analyses and inclusion of validation population year/season in training population, see Supplementary Materials and Methods.

### GS modeling

Seven statistical methods were used for each RYT experiment, including six GS methods: GS + *de novo* GWAS, GS + historical GWAS, RR-BLUP, Bayesian LASSO (BL), Reproducing Kernel Hilbert Spaces (RKHS) and random forest (RF), and one non-GS method: multiple linear regression (MLR). The four non GS+GWAS GS statistical methods were chosen based on their demonstrated success in accurately predicting GEBVs in variety of crops and because they represent the different types of statistical methodologies used to build GS models, that is, linear parametric methods (RR-BLUP, BL), non-linear semi-parametric methods (RKHS), non-linear, non-parametric methods (RF), as well as maximum-likelihood methods (RR-BLUP, RKHS), Bayesian methods (BL) and machine learning methods (RF; Breiman, 2001; Gianola and van Kaam, 2008; Gianola *et al.*, 2009; Heslot *et al.*, 2012; Perez-Rodriguez *et al.*, 2012; Rutkoski *et al.*, 2012; Crossa *et al.*, 2014). For an overview of the methods, see Lorenz *et al.* (2011).

MLR using a subset of markers derived from single marker regressions (MLR) served as our non-GS marker-based prediction control. For each fold, single marker regression was run for all markers and *P*-values determined for each marker by F-test. Note that this is statistical equivalent of a crude GWAS. Linear models were then tested using 1 through the first 100 most significant markers, and the model with the best fit was returned. The returned model was then used to calculate the accuracy for the given fold. For the marker subset experiments where the number of markers in the subset (p) was <100, models were tested using 1 through p markers. Note that our MLR methodology was modified slightly from Spindel *et al.*, 2015 to be more conservative: the validation data were not used in this case to calculate model fit, only the training data were used. This resulted in markedly lower MLR prediction accuracies than those previously reported, particularly for flowering time (Supplementary Materials and Methods).

For the RYT experiments, three CV accuracies were calculated. CV1 accuracies that are also the accuracies reported for the MET data and the CV experiments using marker subsets, were calculated by including the validation year/season in the training population, excluding individuals in the validation fold, for example, for experiment 1, CV1, the training population consisted of data on all training population individuals from years 2009 to 2011 all seasons, as well as the 2012 (the validation season; Supplementary Table S1A). For an explanation of CV2 and CV3, see the Supplementary Materials and Methods 'Inclusion of validation population year/season in training population'.

Narrow sense heritabilities (Table 1) were calculated for each trait in each season, year and site (MET data only) on a per line basis using the rrBLUP package (Endelman, 2011; https://cran.r-project.org/web/packages/rrBLUP/index.html), function mixed.solve, with the least square means for the complete population used as input. The narrow-sense heritabilities were calculated as the additive genetic variance divided by the total phenotypic variance.

#### RR-BLUP+Fixed effects model

When no markers are included as fixed effects, the model is equivalent to standard RR-BLUP (Equation 1), where *y* is the vector of observations, *X* is an incidence matrix for fixed effects containing only a vector of 1s for the intercept, *β* is a vector of fixed effect estimates containing the intercept, *Z* is an incidence matrix for random effects relating individuals to observations and *u* is a vector of random individual effects with *u* ~ N(0, Gσ^2^_μ_), where G is a genomic relationship matrix calculated using all markers (Endelman, 2011). When up to four markers are added to the model as fixed effects, their allele dosages are added as columns to the X matrix and *β* expands accordingly. The markers are then also removed from the calculation of G. All aspects of model fitting otherwise remain the same.


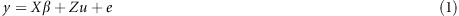


#### Selection of fixed effects for RR-BLUP+fixed effects models

The markers fit as fixed effects were selected from GWAS output, either from a GWAS calculated using the genotype and phenotype data on the individuals in the training population (GS + *de novo* GWAS) or from previously published GWAS data (GS + historical GWAS).

For selection of fixed effects using *de novo* GWAS, the algorithm described below was used for each CV experiment:

1. Run GWAS using Genome-wide Efficient Mixed Model Association (GEMMA) five times, once for each validation fold (Zhou and Stephens, 2012). The input to GEMMA consists of genotype and phenotype data on the individuals in the combined four training folds. Data on individuals in the validation fold is not included in the GEMMA input.
2. Sort the GWAS output by *P*-value (low to high) and perform multiple-test correction using False Discovery Rate (FDR), then bin the SNPs on each chromosome into 500 Kb bins and output the lowest *P*-value SNP in each bin. This step was performed to group SNPs into GWAS peaks—as this was a breeding population with extensive linkage disequilibrium (LD) (Supplementary Figure S2), peaks were large, often spanning ~500 Kb. In other population types, the bin size would need to be modified, most likely decreased, to account for smaller peak size or lower LD. FDR was performed for all SNPs using the R p.adjust() function, method=‘BH' (=benjamini hochberg; Benjamini and Hochberg, 1995).
3. For each fold, load respective GEMMA fold output(s) and save up to the three most significant SNPs (FDR=0.1) for the trait of interest. If no SNPs pass FDR, save only the lowest *P*-value SNP. If only one or two SNPs pass the FDR threshold, save only the SNPs that pass the threshold. Save also the single most significant SNP for flowering time.
4. Test the markers saved from 3. in all combinations and select the markers that, by themselves, constitute the best linear fit using only the training data, that is, select the combination of markers that results in the maximal correlation between the phenotype training data and a prediction resulting from a linear model of the selected marker genotypes. Calculate the average of the FDR corrected *P*-values of the selected fixed effect SNPs=average corrected *P*-value for model.
5. Proceed with model solving and validation phenotype prediction using markers selected in 4.

For the RYT data set, both the 2012 DS and 2012 WS phenotype data were used as the input for GEMMA for each CV experiment. For the MET data set, the 2012 DS RYT data, 2012 WS RYT data, and the 2012 MET phenotype data for the respective validation year, site and season of a given CV experiment were tested as the input to GEMMA for each CV experiment.

Note that we tested models both with and without the lowest *P*-value SNP for flowering time (the 'fourth' marker added to the yield and plant height top three markers) and found that the differences between these models were generally small, although in most cases including the flowering time SNP as a fixed effect improved model accuracy. Given that flowering time alone can shift performance of other agronomic traits, it is always worth controlling for its effect in breeding populations that encounter significant differences in flowering time, as is typical in many rice breeding programs. As SNP selection was performed for each fold, the RR-BLUP+fixed effects models differed slightly in terms of the markers fit as fixed effects by fold.

The above methodology was appropriate in our case because we had already analyzed the GWAS results on the training population data. For a group that wished to replicate our methodology on a new population, we recommend first running GWAS on their training population data to broadly visualize the trait genetic architecture. If a trait has no significant GWAS peaks or peaks that are very near the significance threshold after applying multiple-test correction, the above methodology is not recommended. This GS+GWAS method is intended for traits with one or more medium–large effect QTL segregating in the population. In other words, if random forest is NOT at all predictive for a given trait in a given population, the GS + *de novo* GWAS method presented here will also most likely be unsuitable. For a detailed analysis of the GWAS results by themselves, including candidate gene analysis, see Begum *et al.* (2015).

For the GS + historical GWAS models, the same procedure was used as for the GS + *de novo* GWAS models, except the input to step two of the above algorithm (the GWAS results) was derived from the literature, in our case, from the results of Zhao *et al.* (2011).

The RYT CV results (reported in Supplementary Table S2) were analyzed using analysis of variance (ANOVA) and pairwise Student's *t*-test (that is, Student's *t*-test was performed for each pair of group levels testing only individual comparisons) to determine the effect of statistical method, validation population, and composition of the training population on prediction accuracy. The MET CV results (reported in Supplementary Table S4) were analyzed using ANOVA and pairwise Student's *t*-test to determine the effect of statistical method, combination of sites in the training population, validation site and combination of seasons/years in the training population on prediction accuracy (Supplementary Materials and Methods).

### Analysis of linkage disequilibrium

Pairwise LD matrices were calculated separately for each chromosome using PLINK (http://pngu.harvard.edu/purcell/plink/). Heat maps were generated using Python3 Matplotlib.pcolormesh (http://matplotlib.org) (Supplementary Figure S2).

### CV using SNP subsets

SNP subsets that were chosen to be evenly distributed across the genome (distributed) or chosen at random were selected from the 58,318 genotyping-by-sequencing SNPs with call rates ⩾75% and minor allele frequency ⩾0.05 as described in the Supplemental Materials and Methods. For each marker subset, a genotype matrix for each of the five validation folds was constructed. These genotype matrices were used in conjunction with the phenotype data on the training population individuals to run GEMMA for each subset, for each fold. The GEMMA GWAS results were then used as described in the section 'RR-BLUP + Fixed effects model' above with the marker subset genotype matrices to run GS + *de novo* GWAS CV for each marker subset. RR-BLUP was also run using the marker subsets as a means of comparison.

**Figure 1:**
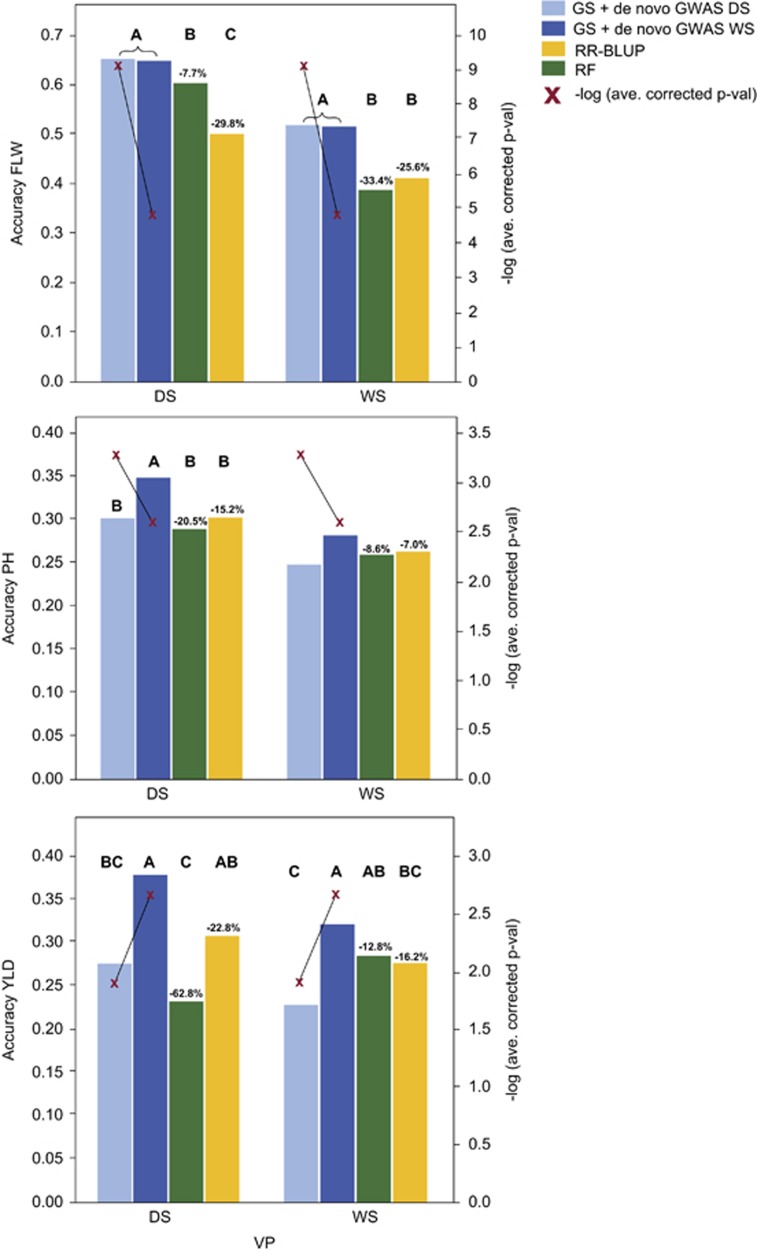
Cross-validation prediction accuracies of flowering time (FLW, top), plant height (PH, middle) and grain yield (YLD, bottom) in the RYT data set, comparing GS + *de novo* GWAS models (blue) to RR-BLUP (yellow) and random forest (RF) (green) models, left axis. Plots show the results using the optimized training population for prediction of each trait in the RYT 2012 dry season (DS) and RYT 2012 wet seasons (WS) (that is, the cross-validation experiment that resulted in the best prediction accuracy for each trait in each validation season, see Supplementary Table S2A). GWAS for the GS + *de novo* GWAS models were run using both the RYT 2012 DS data (light blue) and the RYT 2012 WS data (dark blue). Percent decrease in accuracy of RR-BLUP and RF models versus the average of the two GS + *de novo* GWAS models (FLW), or the GS + *de novo* GWAS WS model are shown over the RR-BLUP and RF bars, respectively. Bars not labeled with the same letter (Pairwise Student's *t*-test) indicate a significant difference in accuracy of the statistical methods across all experiments. Red X's mapped to the right axis=−log * average *P*-value (using the Wald test) of the SNPs fit as fixed effects in the GS + *de novo* GWAS models, after FDR multiple-test correction.

**Figure 2:**
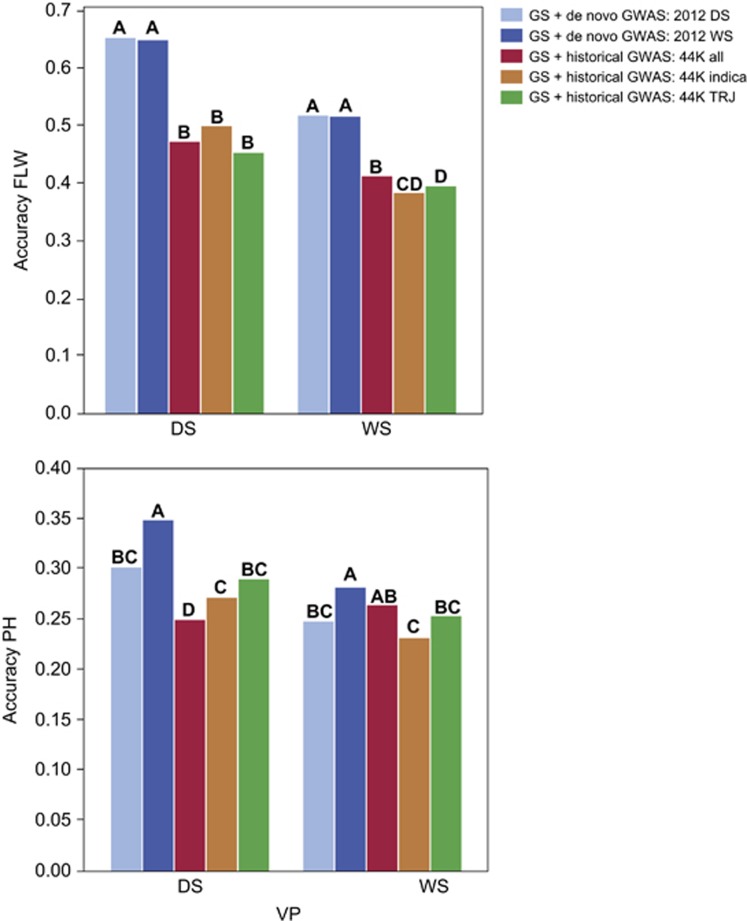
Comparison of GS + *de novo* GWAS with GS + historical GWAS models for flowering time (FLW, top), and plant height (PH, bottom). Graphs shows the results using the optimized training population for prediction of each trait in the RYT 2012 dry season (DS) and RYT 2012 wet seasons (WS; that is, the cross-validation experiment that resulted in the best prediction accuracy for each trait in each validation season; see Supplementary Table S2A). GS + GWAS models differed in the GWAS data used to select the SNPs fit as fixed effects. GS + *de novo* GWAS: 2012 DS (light blue)=*de novo* GWAS using 2012 DS data on training population individuals, GS + *de novo* GWAS: 2012 WS (dark blue) =*de novo* GWAS run using 2012 WS data on training population individuals, GS + historical GWAS: 44K all (red)=previously published (historical) GWAS data were used from Zhao *et al.*, 2011 the 'all subpopulations' results, GS + historical GWAS: 44K indica (burnt orange)= the *indica* subpopulation results from Zhao *et al.* 2011 were used, GS + historical GWAS: 44K TRJ (green)=the *tropical japonica* results from Zhao *et al.* (2011) were used. Bars not labeled with the same letter indicate a significant difference in model accuracies across all experiments.

CV was run using the best performing experiments for each validation season—the same experiments that are reported in Supplementary Table S2A and Figures 1 and 2; see Supplementary Table S3. For the GS + *de novo* GWAS models, the RYT 2012 DS data were used for the phenotype input for flowering time, and the RYT 2012 WS data were used for the phenotype input for plant height and grain yield, as these produced the best GS + GWAS models using the full genotyped data set (Figure 1; Supplementary Table S2A).

**Figure 3:**
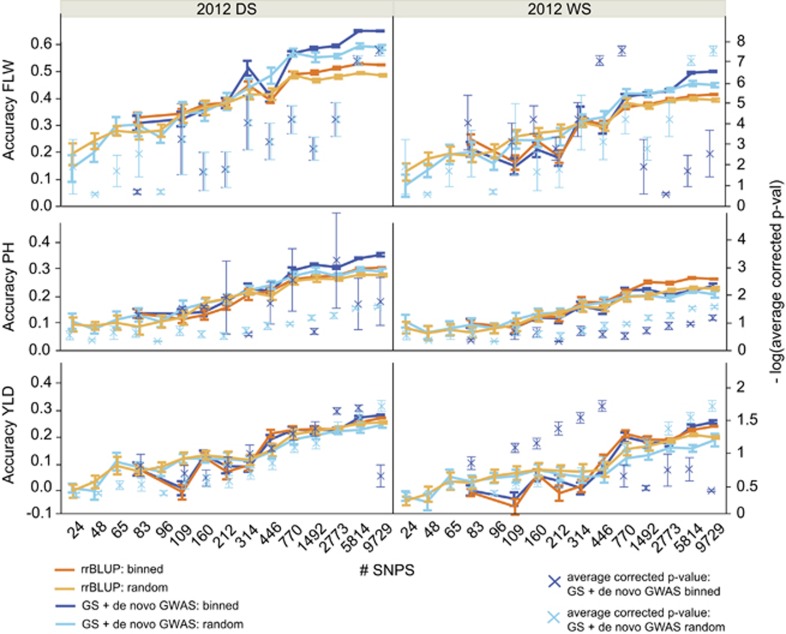
Mean accuracies of cross-validation for prediction of flowering time (FLW, top), plant height (PH, middle) and grain yield (YLD, bottom) in the 2012 dry season (left), and the 2012 wet season (right), using 10 selections of SNP subsets chosen to be either distributed evenly throughout the genome (light shades) or chosen at random (dark shades); left axis. The best performing GS + *de novo* GWAS models (blues), as well as RR-BLUP models (oranges) and previous best performing CV experiments were run for each trait, see Supplementary Table S2A. Right axis (blue X's)=–log * average *P*-value (Wald test) of the SNPs fit as fixed effects in the GS + *de novo* GWAS models, after FDR multiple-test correction. All error bars were construed using 1 s.e. of the mean.

Accuracy was calculated for each of the 10 SNP subsets. A mean accuracy and s.e. for each subset size were also calculated by averaging the CV results of the 10 subsets for each subset size. ANOVA and Pairwise Student's *t* were used to determine the effect of SNP number, SNP type (that is, random or distributed) and statistical method on accuracy (*α*=0.05). Figure 3 plots the accuracy of CV1 for random and distributed SNPs for each validation season and were created using JMP v 12.0 (SAS, Cary, NC, USA).

## Results

Building on recently published studies reporting the results of GWAS and GS in a population of breeding lines from the IRRI irrigated rice breeding program (Begum *et al.*, 2015; Spindel *et al.*, 2015), we investigated the possibility of improving GS prediction accuracy through model refinement by (a) incorporating markers as fixed effects derived from a GWAS performed using the training data set itself (GS + *de novo* GWAS), (b) markers as fixed effects extracted from the literature (GS + historical GWAS) and (c) adding data from multiple environments to the training population. Two data sets consisting of 108 005 SNPs on ~363 elite irrigated rice breeding lines were used, a RYT data set consisting of 4 years of data (2009–2012), two seasons per year (dry and wet), taken at a single site at IRRI in Los Baños, Philippines, and a multi-environment trial (MET) data set, collected over two years (2011–2012, DS and WS per year) at four locations in 2011 and eight locations in 2012 (Table 1; Methods). For both data sets, we focused on prediction of three traits that differed in their genetic architecture, as shown by the results of GWAS run on the same data sets: flowering time (FLW), a trait controlled by a few large effect QTL, grain yield (YLD), a trait controlled by many small effect QTL, and plant height (PH), a trait controlled by both large and small effect QTL (Begum *et al.*, 2015; Spindel *et al.*, 2015). In the RYT data set, narrow-sense heritabilities ranged from 0.27 to 0.44 for FLW, 0.24 to 0.39 for PH and 0.07 to 0.32 for YLD depending on the year and season, while in the MET data set, heritabilities range from 0.05 to 0.64 for FLW, 0.0 to 0.52 for PH, and 0.0 to 0.74 for YLD, depending on the year, season and site (Table 1).

### Use of fixed effects extracted from the training population to improve accuracy of GS models (GS + *de novo* GWAS)

One means of boosting GS prediction accuracies is to incorporate additional genomic and/or biological information, such as that revealed in a GWAS, into the GS model. To exemplify how such integrated models can improve performance, we performed CV using the RYT data set. For all CV experiments, we developed RR-BLUP models in which 1–3 of the most significant SNPs identified by fold-specific GWAS (run using the 2012 phenotype data on individuals in the training population) were included as fixed effects (model=GS + *de novo* GWAS; Supplementary Table S2, Methods section). GWAS were run using both the 2012 DS and 2012 WS data, and for every cross-validation experiment, two models were tested, one in which the most significant SNPs (binned on a 500 Kb basis) from the DS GWAS were tested for incorporation as fixed effects, and one in which the most significant binned SNPs from the WS GWAS were tested. The results of the GS + *de novo* GWAS were compared with 1. GS + historical GWAS models, in which the markers fit as fixed effects were selected from previously published GWAS data, and 2. the five other genotype-based prediction methods previously tested in this population: RR-BLUP without any fixed effects, RKHS, random forest (RF), Bayesian LASSO, and multiple linear selection (MLR; Methods section; Spindel *et al.*, 2015). The results of all experiments are given in Supplementary Table S2B.

The effect of training population composition and validation population on prediction accuracy in this population has been discussed elsewhere (Spindel *et al.*, 2015), and is presented in Supplementary Table S2B for the sake of completeness. We focus here on the effect of statistical method on accuracy in the best performing cross-validation experiments, that is, the combination of training years/seasons that, on average, produced the best prediction accuracies. Across all traits and experiments, the most accurate statistical methods of those tested were the GS + *de novo* GWAS models (Figure 1; Supplementary Table S2).

For the best performing CV experiments for each trait and season (Supplementary Table S1A) the GS + *de novo* GWAS using the 2012 WS data as input to the GWAS outperformed simple RR-BLUP in all cases (Figure 1; Supplementary Table S2). The percent improvement ranged from ~29.8% for FLW in the DS, to ~7.0% for PH in the WS (Figure 1; Supplementary Table S2A). Furthermore, for all traits and seasons, the GS + *de novo* GWAS model (using the 2012 WS data for PH and YLD) was also the most accurate overall, outperforming RF, the next best performing model for some trait x season combinations (Figure 1; Supplementary Table S2A). These gains were generally modest, ranging from ~12.8% for YLD in the WS to ~7.7% for FLW in the DS. Although not all differences were significant (Figure 1; Supplementary Table S2), these results demonstrate that identifying markers that tag important genes and adding them as fixed effects to GS models can enhance GEBV prediction, sometimes markedly. In no case did adding fixed markers identified chosen based on the GWAS of the 2012 WS data decrease accuracy relative to RR-BLUP or any other tested statistical method (Figure 1; Supplementary Table S2).

It is not entirely clear why the 2012 WS data were a more effective source of fixed SNPs for plant height and grain yield than the 2012 DS data, however, a few possibilities exist. For YLD, the best explanation is that the most significant SNPs identified using the 2012 WS data were considerably more significant after multiple-test correction than those identified using the 2012 DS data (Figure 1; Supplementary Table S2). Although all SNPs included in all models were significant by FDR=0.1, if the significance threshold were to be raised slightly, some SNPs would have been dropped (Figure 1). Furthermore, for YLD, decreased average corrected *P*-value of the SNPs fit as fixed effects correlated with decreased prediction accuracy. This was not the case, however, for plant height, where all SNPs fit as fixed effects were well above the FDR threshold, and where using the 2012 DS data as the GWAS input resulted in lower GS prediction accuracies, but higher average corrected *P*-values of fixed effect SNPs. It thus does not seem likely that the significance of the GWAS results was a contributing factor in the improved performance of the 2012 WS data for plant height (Figure 1). Instead, it is possible that stochasticity in the data resulted in more informative QTL being identified in the WS than in the DS, or that increased disease pressure in the WS resulted in more reliable prediction from wet to dry than vice versa. Overall, the results suggest that when there are highly significant peaks identified in a GWAS, adding markers that tag these GWAS peaks as fixed effects in an RR-BLUP+ fixed effects models improves accuracy over those obtained from more complex models like RF.

### Use of fixed effects extracted from the literature to improve accuracy of GS models

To determine whether significant GWAS-SNPs identified for the same traits but using different germplasm would be equally useful as fixed variables in our GS models, we compared prediction accuracies for flowering time and plant height of the above GS + *de novo* GWAS models to three additional RR-BLUP+fixed effects GS models in which the fixed SNPs were selected using GWAS data from Zhao *et al.* (2011). (There were no previously published GWAS data available for YLD, so it was not possible to compare the results for this trait.) The three additional models tested utilized SNPs identified by GWAS in a rice diversity panel representing (1) the *indica* subpopulation, (2) the *tropical japonica* subpopulation, and (3) the all, or combined subpopulations (Zhao *et al.*, 2011). As our training population consisted of only *indica* individuals (Methods section; Spindel *et al.*, 2015), this allowed us to test, in addition to the effect on model accuracy of using previously published GWAS results, the effect of using previously published GWAS results derived from individuals from the same subpopulation undergoing selection in breeding, versus the effect of using GWAS results derived from individuals from different subpopulations than the one undergoing selection (Figure 2; Supplementary Table S2).

For flowering time, using GWAS results derived from the training population proved to be significantly more accurate than using any of the historical GWAS results. For the DS, it made little difference which historical GWAS data were used, all data sets performed badly. For the WS, the all subpopulation results were significantly better than using either the *indica* only or *tropical japonica* only results, but again, all were significantly worse than using either the 2012 DS or 2012 WS data. (Figure 2; Supplementary Table S2). For plant height in the DS, using the 2012 WS data resulted in significantly more accurate GS models than using the historical GWAS data, but for the WS, the *all* subpopulation historical data performed about as well as the 2012 WS data (Figure 2; Supplementary Table S2).

In essence, these results suggest that in some cases, the researcher may get lucky when utilizing previously published results, that is, in some cases, historical GWAS results will be relevant to a given breeding population, as was the case for plant height in our WS. In other cases, however, such as for flowering time in this breeding population, SNPs identified in previously published GWAS will not be relevant to a given population, and will thus decrease accuracies relative to simple RR-BLUP models. Regardless of whether previously published results might perform as well as *de novo* results, in our experiment, the previously published GWAS data never improved model accuracy over the GS + *de novo* GWAS models, thus, there appears to be no reason to pursue this strategy (Figure 2; Supplementary Table S2).

### Number of markers and GS accuracy

We also tested the accuracy of the best performing GS + *de novo* GWAS models at decreasing numbers of genome-wide markers. (GWAS were also run with the decreasing number of markers as it is fully integrated into the RR-BLUP+fixed effects GS model). Consistent with previous results, we found that ~5000 SNPs were as effective for prediction as the full marker set of 108 005 SNPs (Figure 3; Supplementary Table S3). After that, accuracies began to decrease significantly, regardless of whether the genome-wide SNPs were evenly distributed or not. Average corrected *P*-value of the SNPs fit as fixed effects generally decreased in tight correlation with decreasing SNP number. This is to be expected given that a smaller pool of SNPs also means a smaller chance of identifying a SNP in high LD with a QTL of interest.

These results suggest that it may be possible to design smaller fixed SNP arrays for GS that reduce genotyping costs and increase turn-around time (Thomson, 2014; Yu *et al.*, 2014). Heat maps showing the extent of linkage disequilibrium across each chromosome are given in Supplementary Figure S2.

### GS model refinement using multi-environment data

To evaluate the effect of multi-environment data on GS model accuracy, we ran five-fold cross-validation using the IRRI METs consisting of two years of yield data (2011–2012) over two seasons/year (dry and wet) taken at IRRI headquarters in Los Baños and at four additional sites in SE Asia during 2011, and at eight additional sites in 2012. Because of typhoons and pests in 2011, data were available for only two of four sites in the DS and one of four sites in the WS, while in 2012, data were available on only a subset of lines due to breeding program progression (Table 1). The unbalanced nature of the data set is typical of historical public breeding program data, so it is worth determining the value of such data sets, even if they are statistically non-ideal.

The composition of the training population was varied in terms of sites, seasons, and years, as is typical for MET data (Supplementary Table S1B). We tested GS + *de novo* GWAS and RR-BLUP models for all traits, as well as GS + historical GWAS and RF models for flowering time and plant height. For the GS + de novo GWAS models we used as GWAS inputs the results of GWAS run on the validation site and year (on the individuals in the training population), as well as the RYT 2012 DS and 2012 WS GWAS results used previously.

Validation site, validation season, the combination of sites in the training population, and the statistical method all contributed significantly to prediction accuracy in the MET data set (Supplementary Table S4). The combination of sites in the training population was of particular importance. Across all traits, two groupings consistently produced the highest mean prediction accuracies for the sites in each group, a group consisting of the sites with southernmost latitudes: Bohol, Midsayap, Sri Lanka, and Agusan, and a group consisting of the sites with northernmost latitudes plus Agusan: Nueva Ecija, Isabela, Los Baños/IRRI and Agusan (Table 2). Agusan was unusual; it could improve the prediction at the northern sites despite the fact that it is in the southern Philippines and is best predicted by the southern group (Table 2). One possible explanation for this may be the unusually wet 'dry season' at Agusan, which could mean that individuals that do well in Agusan are likely to also do well almost anywhere else in SE Asia.

Historically, these eight sites have been treated as representative of the key rice growing regions in the Philippines/SE Asia, or as a single target population of environments for breeding purposes. Our results here indicate that splitting these sites into the 'northern' and 'southern' groups, with Agusan included in both groups, improves prediction accuracies up to 10-fold. Results were especially significant for prediction of grain yield (Table 2).

**Figure 4:**
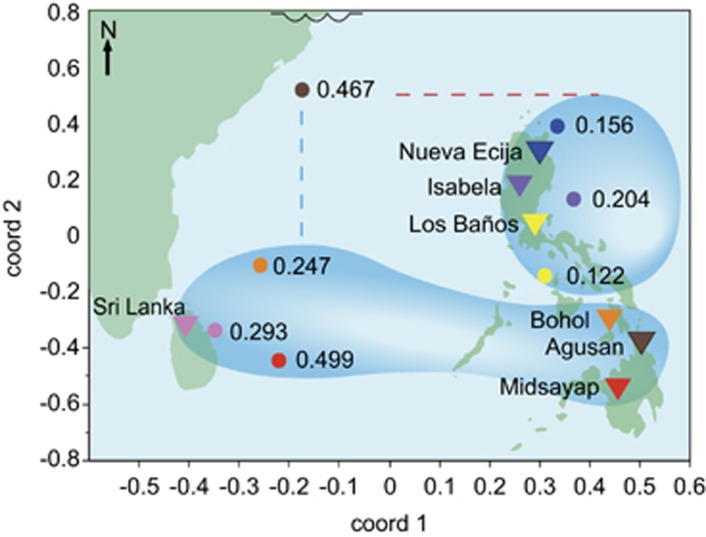
Multi-dimensional scaling (MDS) analysis of the distance matrix of the MET adjusted 2012 wet season yield data overlaid on a map of the sites. Triangles=locations of sites, Circles= MDS points, site locations and MDS points have corresponding colors. Values= highest grain yield CV accuracy obtained for that site *using the displayed site grouping*, bubbles= groupings of sites that produced the highest mean prediction accuracies at those sites. Agusan is clearly an outlier—while it geographically belongs to the southern group and is best predicted by southern group, blue dashed line, it can also improve prediction accuracies of northern group, red dashed line. Squiggle at the top of the plot indicates a break in longitudinal map space.

These results reflect the correlation of phenotypes within the northern and southern groupings; Figure 4 shows a multi-dimensional scaling analysis using the 2012 WS data for grain yield, in which the points can be superimposed on a geographical map of the sites. The phenotypic correlation of traits measured in Agusan with both the northern and southern sites offers an explanation for why inclusion of Agusan in both groups improves prediction accuracies of all sites (Figure 4; Supplementary Figure S1). These results are consistent with current breeding practice within the Philippines—the Philippines national variety release system now makes region-specific varietal recommendations. Notably, for all but three site x trait combinations, the highest mean site grouping was also significantly better than using only the validation site, evidencing the benefit of utilizing correlated multi-environment data for genome-wide prediction.

**Figure 5:**
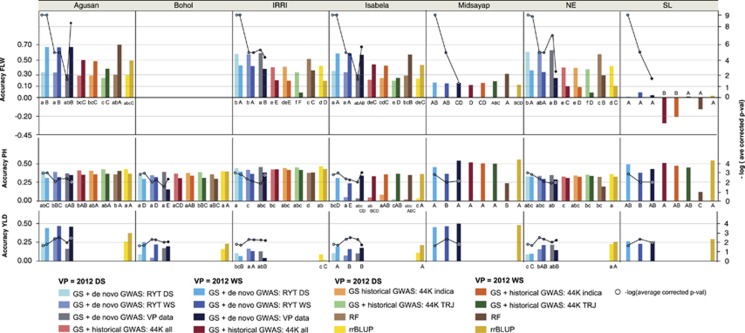
Cross-validation prediction accuracies of flowering time (FLW, top), plant height (PH, middle), and grain yield (YLD, bottom) using multi-environment (MET) data. Data show the best overall MET accuracies obtained for each trait in each validation season, the 2012 dry season (DS; light shades) and the 2012 wet season (WS; dark shades), and validation site, left axis (Supplementary Table S4A). Accuracies are compared for GS + *de novo* GWAS models using, as GWAS input, the RYT 2012 DS GWAS results (blue bars), the RYT 2012 WS GWAS results (purple bars), and GWAS run using the validation site and season (gray/black bars) to RR-BLUP results (yellow bars), and for FLW and PH only, the GS + historical GWAS results (red, orange, and green bars for 44K all, 44K *indica*, and 44K *tropical japonica* results, respectively), and random forest (RF) results (brown bars). Bars not labeled with the same lower case letter indicate a significant difference in the performance of statistical methods across all experiments where the validation population=2012 DS, bars not labeled with the same capital letter indicate a significant difference in the performance of statistical methods across all experiments where the validation population=2012 WS. Circles mapped to right axis=−log * average *P*-value (Wald test) of the SNPs fit as fixed effects in the GS + *de novo* GWAS models, after FDR multiple-test correction.

The maximum prediction accuracies obtained for each site and trait using training populations containing data from the site groupings described above are shown in Figure 4, while the highest overall prediction accuracies for each site x season x trait combination are shown in Figure 5. In some cases, higher accuracies were obtained using different combinations of sites in the training population than the northern and southern groupings, despite these groupings producing the best average accuracies. Given that many sites and seasons had low heritabilities and low accuracies overall, we must be cautious when drawing conclusions regarding use of statistical method from this data set. In general, the RYT results are considered more reliable. A few trends are, however, apparent. For the majority of site x season combinations for flowering time, GS + *de novo* GWAS was clearly the best statistical method across experiments. For the best overall experiments for grain yield (Supplementary Table S4A), the GS + *de novo* GWAS models generally outperformed the other statistical methods, but for most sites and seasons, the difference was not significant across experiments. For plant height, by contrast, RR-BLUP was generally the best performing statistical method for the best experiments, but again, in all but two cases, the difference in accuracy when using RR-BLUP versus the other statistical methods was not significant across experiments (Figure 5; Supplementary Table S4). In general, using the RYT 2012 DS and RYT 2012 WS data as the GWAS input for the GS + *de novo* GWAS models resulted in better prediction accuracies than using the validation data, most likely as a result of the higher quality of the RYT data and generally more significant *P*-values (Figure 5; Supplementary Table S4).

The prediction accuracies themselves ranged for flowering time from a high of 0.70 to a low of −0.34 for Sri Lanka in 2012 WS. For plant height, the highest overall accuracies ranged from 0.55 for Midsayap in 2012 WS, the best, to 0.01 for Isabela in the 2012 DS, the worst. For yield, the highest was 0.50 for Midsayap in the 2012 WS and the lowest was 0.09 for Los Baños/IRRI in the 2012 WS. These large differences in the maximum accuracies obtained at different sites and in different seasons are largely explained by the amount of data available for inclusion in the training population, given the high frequency of natural disasters in the region, by the degree of correlation between years and sites, and to a lesser extent, by the trial heritability and statistical method. For example, prediction accuracies at Agusan were generally high because training data were available from 3 seasons (2011 WS, 2012 DS and WS), and Agusan was well correlated with the other southern sites (Figures 4 and 5; Supplementary Tables S4). Isabela, on the other hand, had very low accuracies for prediction in the 2012 DS, very low trial heritabilities (0 and 0.7 for PH and YLD, respectively, and the site/season was not correlated with any other site or season, including itself in the WS, all of which evidence that the phenotyping data for this site and season were compromised in some way (Table 1, Figure 5; Supplementary Figure S1, Supplementary Table S4). The highly negative accuracy for flowering time at Sri Lanka, on the other hand, was obtained using the GS + historical GWAS results, which could indicate that in some cases using historical GWAS results on a population in which they are not relevant can result in negative correlation accuracies, possibly as a result of differences in linkage phase between the tagged SNP and QTL in the two populations, or as a result of epistasis.

Conducting multi-location field trials is a challenging and massive logistical operation that, in the public sector, is also under-resourced. GS, on one hand, appears to be more sensitive to low-quality data and/or a poorly defined target population of environments (TPE) than phenotypic selection (Heslot *et al.*, 2015), and our results indeed highlight a strong need for high-quality phenotype data. They also suggest, however, that collecting higher quality data at fewer sites could enable good genome-based predictions at other correlated sites, an observation in agreement with multi-environment experiments performed in maize and wheat (Burgueño *et al.*, 2012; Heslot *et al.*, 2013; Crossa *et al.*, 2014; Zhang *et al.*, 2015). By focusing on quality data at a few key, representative environments, it could thus be possible to improve gain-from-selection across an entire region.

Ultimately, GS prediction accuracies suffer when badly correlated environments are combined due to GxE effects, that is, the fact that a given variety may perform well in one environment, but poorly in another. Even in a traditional pedigree breeding scheme, it is important to accurately group common environments in order to avoid deleterious GxE effects in released varieties. An alternative solution to utilizing multi-environment data is to explicitly model GxE in the GS model, as has recently been shown effective in wheat (Lopez-Cruz *et al.*, 2015). This strategy is likely to be more useful with cleaner and more complete data sets, but warrants further research for the refinement of rice GS models.

### Use of GS+GWAS to expedite the introduction of novel genetic variation into elite breeding populations

GS, while new in application, is conservative in breeding effect. Genome-wide prediction models are trained only on alleles and genetic diversity present in a given population, and as such, the alleles selected for by GS are those already known to contribute to good performance. In rice and many other crops, however, new diversity and GxG interactions are important sources of trait improvement that are essential for enhancing genetic gain (Li *et al.*, 1997; Thomson *et al.*, 2006; McCouch *et al.*, 2012; McCouch *et al.*, 2013). As a result, to make GS work for rice breeding, it is necessary to 1) identify and tap sources of novel variation, and 2) to develop methods that will introduce these favorable new alleles into adapted varieties while retaining the highly productive allele combinations that are the foundation of food production.

**Figure 6:**
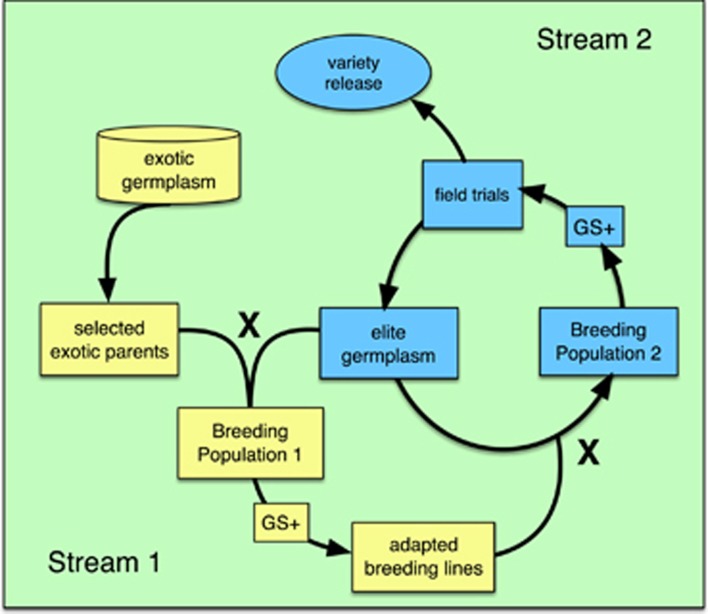
Diagram of proposed two-stream GS breeding program. Stream 1 (yellow boxes) consists of pre-breeding, in which favorable alleles from exotic germplasm are introduced into adapted germplasm. Exotic parents are crossed with elite germplasm to develop Breeding Population 1. Selection of individuals from Breeding Population 1 is performed using a combination of GS + *de novo* GWAS models (GS+), in which the exotic QTL are fit as fixed effects, and phenotype. The training population GS would be a subset of breeding population 1, that is, a fraction of breeding population 1 would be both genotyped and phenotyped, while the rest of breeding population 1 would be genotyped only. Adapted materials from Breeding Population 1 are crossed into Breeding Population 2 (Stream 2, blue boxes) where they are further refined using GS + *de novo* GWAS models, where the fixed effects would include valuable QTL identified based on GWAS performed in Breeding Population 2, the exotic QTL from Stream 1, or any other large effect QTL a breeder might normally target for trait improvement. Output from Stream 2 can be advanced toward variety release or fed back into Stream 1 to serve as parents for further crossing and population development.

To address this issue, we propose a two-stream/two-part GS breeding schema in which under-utilized germplasm is systematically incorporated into a GS breeding pipeline to test for and predict the presence of new, highly effective allele combinations (Figure 6). Stream 1 consists of pre-breeding, in which new alleles sourced from diverse germplasm are sequentially introduced into a population of adapted material. After several rounds of backcrossing and recombination, necessary to break linkages after the initial F_1_ cross between un-adapted and adapted material, GS + *de novo* GWAS models would be used to increase the frequency of desirable exotic QTL while simultaneously selecting against alleles from un-adapted material that conferred negative effects. Such desirable QTL would be identified by the *de novo* GWAS, and quickly fixed as a direct result of the GS + *de novo* GWAS approach. Stream 2 continues the process of refining and improving existing elite material, and by feeding the output of Stream 1 into Stream 2, the genetic base of modern varieties would be expanded. Valuable QTL in the Stream 2 breeding population would also be identified via *de novo* GWAS and fit as fixed effects. These fixed effects could also include the exotic QTL from Stream 1, and any other large effect QTL a breeder might wish to target for either positive or negative selection.

The above approach would enable the breeder to learn directly from data on new and diverse germplasm and make rapid genetic gain in a way that would not be possible using simple RR-BLUP models, as it is the GS + *de novo* GWAS strategy that makes it possible to extract the information necessary for fixing valuable exotic alleles during model development as well as enhancing prediction accuracy. Furthermore, no prior knowledge about genes, QTL, or gene networks is required. Thus, while it is interesting to extrapolate as to which genes are involved in a given biological process and to compare new GWAS results to those previously published, the breeder is not required to do so, and is not encumbered by the need to identify causal polymorphisms or candidate genes underlying potentially large regions of significance *a priori*. As a result, this approach empowers the breeder to move forward immediately with selection in a breeding population based on GEBVs with the knowledge that GEBVs are derived *de novo* from relevant breeding material, trait evaluations, and target environments, in keeping with the objectives and realities of the breeding program at hand.

## Discussion

GS, or genome-wide prediction, has been heralded as a strategy that can help increase the rate of genetic gain in plant and animal breeding without prior knowledge of the genes or QTLs underlying agronomic traits (Rutkoski *et al.*, 2012; Asoro *et al.*, 2013; Massman *et al.*, 2013a; Crossa *et al.*, 2014; Beyene *et al.*, 2015; Onogi *et al.*, 2015; Spindel *et al.*, 2015; Zhang *et al.*, 2015). Our work suggests that using biological knowledge about genotype–phenotype associations, as demonstrated by the GS+ *de novo* GWAS model results presented here, can improve the prediction accuracies of GS in rice. Given the amount of basic biological information available for many crop species today, and for rice in particular, bypassing the opportunity to integrate genic and QTL information into GS models means forfeiting a significant component of model accuracy. However, integrating inaccurate or inappropriate priors or fixed effects can have a negative impact on GS models.

In this study, we aimed to develop a generically useful approach to identifying SNPs to include in GS prediction models as fixed effects that would take advantage of the matrix of genotypic and phenotypic data already generated for the GS study, and would not require independent, *a priori* information about known functional markers for traits of interest. Previous work has relied on the use of historical information to identify markers that can be fit as fixed effects (Bentley *et al.*, 2014; Bernardo, 2014; Owens *et al.*, 2014; Rutkoski *et al.*, 2014; Zhang *et al.*, 2014; Zhao *et al.*, 2014; Lipka *et al.*, 2015). Although these examples provide evidence that introducing fixed effects can improve the prediction accuracy of GS models, this study is the first to quantify the benefit of including markers as fixed effects in RR-BLUP models for a rice breeding population, and is also the first, to our knowledge, in which the identification of SNPs to include as fixed effects in the GS model was accomplished based entirely on *de novo* GWAS (information from GWAS performed on the breeding population undergoing selection) an easier and more stream-lined procedure than delving into the literature to ensure that the GWAS markers tagged previously reported genes of large effect.

In a study by Zhang *et al.* (2014), the authors used the results of previously published GWAS studies to improve GS prediction in dairy cattle and rice, but rather than performing a GWAS using data from the training (breeding) population under selection, as we have done here, they used generic models for each species. Although that strategy may be effective for dairy cattle, which are characterized by an extremely narrow genetic base and highly uniform production environments, it was not expected to prove as effective for plant species such as rice where deep population structure and highly variable production environments create the need to derive population- and environment-specific fixed variables (Zhao *et al.*, 2011; Guo *et al.*, 2014; Heslot *et al.*, 2015; Supplementary Note). To confirm this hypothesis, we compared our models in which markers fit as fixed effects were selected based on the results of GWAS performed on the breeding population to models in which SNPs were selected as fixed effects based on previously published GWAS data, both when subpopulation did and did not match the individuals undergoing selection. In no case did using previously published GWAS data significantly improve accuracy over using *de novo* GWAS, and in the majority of cases, the GS + historical GWAS models were significantly worse than the GS + *de novo* GWAS models (Figures 2 and 5; Supplementary Tables S2). In a few rare cases, the GS + historical GWAS models even resulted in negative prediction accuracies, suggesting that extreme care would need to be taken if perusing this strategy in rice populations.

While the GS + *de novo* GWAS models performed well overall, the data used as input to GS + *de novo* GWAS models did have a significant effect on prediction accuracy for 2/3 traits examined, that is, plant height and grain yield. The grain yield case is of particular interest, because the difference in performance of the GS + *de novo* GWAS models that used as GWAS input the 2012 WS data performed significantly better than the GS + *de novo* GWAS models that used the 2012 DS data as input across experiments for both validation seasons. As discussed in the results, this observation is best explained by the difference in FDR corrected *P*-value of the most significant SNPs resulting from the two GWAS. From these results, we conclude that the utility of GS + *de novo* models is noteworthy when the *de novo* GWAS results identify highly significant SNPs, but may not improve accuracies significantly if GWAS *P*-values are borderline. Based on this population, we would recommend utilizing the GS + *de novo* GWAS model over alternatives such as RR-BLUP or RF when the -log (FDR corrected *P*-values of the most significant SNPs) ⩾2.0. Finally, it is worth noting that as with any GWAS-based methodology, controlling for subpopulation structure in the population is essential, otherwise, associations may be spurious and lead to decreased prediction accuracies.

Our results indicate that the GS + *de novo* GWAS approach will be successful for rice breeding within the boundaries described above. The approach should allow breeders to extract information from the training population and, simultaneously, to learn which regions of the genome are significantly associated with traits of interest in their material. The breeder can then use that information to improve the accuracy of their GS models. Consequently, breeding programs can operate with significant autonomy, unencumbered by the need to identify genes or QTL underlying traits of interest, as was the case for MAS and many previously tested GS plus fixed effect models. Furthermore, the accuracy of our GS + *de novo* GWAS models can be iteratively improved, as information from subsequent training populations is continuously fed back into the model to improve model fit and accuracy.

How, then, does the GWAS and GS information generated by a breeding program intersect with published information about genes, QTLs, expression networks, physiological pathways, developmental phenotypes, etc. As breeding programs increasingly invest in the sequencing, genotyping and large scale phenotyping of populations and germplasm resources in environments that are relevant to the development of new, commercial crop varieties, they will build important bridges that enable data and information to flow between the world of basic biological research and that of applied or translational science, leading to the development of collaborations and research networks that will help bring these two worlds closer together. Scientists interested in population biology, molecular genetics and gene discovery will help discover and characterize genes and alleles associated with phenotypes of interest to the plant breeder, providing useful tools and insights about natural variation that can be help breeders, agronomists, gene bank managers, physiologists and ecologists to better manage natural variation and to generate sustainable systems capable of producing the food, feed, fiber and fuel needed for a growing world population.

Another important source of phenotype variation in breeding populations is derived from the environment (*V*_pE_) and Genotype x Environment (GxE) interactions (*V*_pGE_). Previous GS experiments in wheat and barley have found that multi-environment GS models can lead to improved accuracies by borrowing information from correlated environments (Burgueño *et al.*, 2012; Heslot *et al.*, 2013; Crossa *et al.*, 2014). To see if we could likewise improve accuracies using multi-environment data in rice, we performed CV using phenotype data collected at an additional seven sites in SE Asia. When data were well correlated across sites, GS accuracies increased up to 60 times that of single site models (Table 2). However in other cases, when uncorrelated sites were combined in the training populations, accuracies essentially plunged to zero. These results emphasize the importance of accurately defining a TPE before attempting to perform GS using multi-environment data.

It has been suggested that defining a TPE is of greater importance when performing GS than phenotypic selection because the results of 'bad data' going into a GS model can have long-term impacts on breeding program gains (Heslot *et al.*, 2015). Our MET results strongly indicate two groupings of environments among the eight sites tested here—a ‘northern' TPE consisting of the sites at Nueva Ecija, Isabela, Los Baños and Agusan, and a 'southern' TPE consisting of the sites at Agusan, Midsayap, Bohol and Sri Lanka (Table 2, Figures 4 and 5; Supplementary Table S4). Hai Dong, Vietnam, was an outlier that should not be included in either group (Supplementary Figure S1). Finally, the results highlight the extreme variability in phenotype data quality across sites, seasons and years, and the strong need for consistent phenotype quality to for GS to be implemented effectively. This need is all the more important in the tropics where extreme weather events (for example, typhoons) may eliminate one or more sites/seasons of data. One useful strategy may be to collect higher quality data at a few, key, representative sites and then predict performance at other correlated locations.

The final question is when and how to incorporate knowledge about GEBVs derived from GS models into applied breeding pipelines. GEBVs offer plant breeders an opportunity to integrate knowledge about quantitative trait performance in their breeding populations early in their breeding pipelines, while traditionally, this knowledge is only available at the end of a multi-environment/multi-year replicated yield trial. Because the GEBV's summarize information that's derived directly from the breeders' fields, it is, in essence, simply a more objective measure of what a breeder practicing phenotypic selection would use as the basis for making selections. GS also provides that information in advance so the breeder can use it to select the lines that merit more in-depth phenotypic evaluation. Thus, while the use of GEBVs may differ depending on the trait, breeder and the kind of training population from which the estimated breeding values were derived, they provide the breeder with an additional selection criterion that can be used to increase the rate of genetic gain.

The greatest and most accessible source of untapped genetic diversity for plant improvement can be found in the wild and cultivated accessions housed in the world's gene banks. Although there have been many efforts to screen gene bank material to identify individuals carrying specific traits of interest, it has not been feasible to screen large numbers of accessions for favorable alleles that contribute to useful quantitative variation (McCouch *et al.*, 2012), particularly where the phenotype of the donor germplasm is not obviously superior. While we do not report doing this here, the potential to use GS models, in combination with high throughput genotyping, makes it possible.

The first step would be to genotype the gene bank materials to facilitate a systematic sampling and exploration of the variation in the gene bank. The second would be to incorporate a diverse selection of gene bank materials into a GS breeding pipeline, generating advanced backcross or possibly multi-parent advanced generation inter-cross populations in adapted genetic backgrounds (pre-breeding). To determine which lines from these populations to use as parents in future crossing and population development, training populations representing these new populations would be genotyped and phenotyped as the basis for GWAS to identify new and highly effective allele combinations that were predictive of top-performing offspring (that is, GWAS in breeding panels/training populations). The significant GWAS-SNPs would then be used as fixed variables in GS models (previously developed to facilitate the selection of adapted, elite breeding materials) to facilitate the efficient selection of lines from the new populations that carried favorable exotic alleles in the genetic background of elite lines with high GEBVs.

In summary, here we demonstrate that by incorporating information from GWAS and correlated sites into GS models, prediction accuracies can increase to the point that genotyping and performing GS is more cost-effective than planting and phenotyping additional yield trials (Spindel *et al.*, 2015). With the addition of novel diversity from gene banks, genomics-assisted selection should be a transformative strategy for rice improvement.

## Data archiving

The genotype data and RYT phenotype data are available from the Dryad Digital Repository: http://dx.doi.org/10.5061/dryad.7369p. MET phenotype data will be uploaded to rice diversity.org on article publication and are also available from the Dryad Digital Repository: http://dx.doi.org/10.5061/dryad.vv28j.

## Figures and Tables

**Table 1:** Summary of the two data sets used in this study

*Data set name*	*Site*	*Year*	*Season*	*Lines w/ data*	*Missing data*	*Phenotypes of interest*	h^2^
RYT	LB RYT plot	2009	dry	114	218	FLW, PH, YLD	F: 0.28 P: 0.24 Y: 0.31
RYT	LB RYT plot	2009	wet	163	169	FLW, YLD	F: 0.32 Y: 0.19
RYT	LB RYT plot	2010	dry	166	166	FLW, PH, YLD	F: 0.27 P: 0.35 Y: 0.07
RYT	LB RYT plot	2010	wet	209	123	FLW, PH, YLD	F: 0.38 P: 0.26 Y: 0.13
RYT	LB RYT plot	2011	dry	327	5	FLW, PH, YLD	F: 0.36 P: 0.39 Y: 0.27
RYT	LB RYT plot	2011	wet	328	4	FLW, PH, YLD	F: 0.39 P: 0.26 Y: 0.13
RYT	LB RYT plot	2012	dry	325	7	FLW, PH, YLD	F: 0.44 P: 0.35 Y: 0.32
RYT	LB RYT plot	2012	wet	324	8	FLW, PH, YLD	F: 0.33 P: 0.30 Y: 0.31
MET	I	2011	dry	323	9	FLW, PH, YLD	F: 0.38 P: 0.15 Y: 0.06
MET	NE	2011	dry	327	5	FLW, PH, YLD	F: 0.30 P: 0.23 Y: 0.16
MET	A	2011	wet	323	9	FLW, PH, YLD	F: 0.43 P: 0.30 Y: 0.23
MET	A	2012	dry	202	130	FLW, PH, YLD	F: 0.05 P: 0.47 Y: 0.26
MET	B	2012	dry	203	129	FLW, PH, YLD	F: 0.09 P: 0.14 Y: 0.16
MET	I	2012	dry	203	129	FLW, PH, YLD	F: 0.13 P: 0 Y: 0.07
MET	LB MET plot	2012	dry	203	129	FLW, PH, YLD	F: 0.39 P: 0.32 Y: 0
MET	NE	2012	dry	203	129	FLW, PH, YLD	F: 0.24 P: 17 Y: 0.08
MET	A	2012	wet	203	129	FLW, PH, YLD	F: 0.45 P: 0.09 Y: 0.05
MET	B	2012	wet	203	129	PH, YLD	P: 0.23 Y: 0.13
MET	I	2012	wet	203	129	FLW, PH, YLD	F: 0.31 P: 0.52 Y: 0.13
MET	LB MET plot	2012	wet	203	129	FLW, PH, YLD	F: 0.34 P: 0.39 Y: 0.06
MET	NE	2012	wet	203	129	FLW, PH, YLD	F: 0.19 P: 0.08 Y: 0.13
MET	M	2012	wet	53	279	FLW, PH, YLD	F: 0.64 P: 0.28 Y: 0.74
MET	SL	2012	wet	53	279	FLW, PH, YLD	F: 0.41 P: 0.40 Y: 0.26
MET	V	2012	wet	53	279	FLW, PH, YLD	F: 0.38 P: 0 Y:0.14

(1) the Replicated Yield Trial (RYT) data set, consisting of 4 years of data (2009–2012), two season per year (dry and wet), taken at 1 plot at IRRI, in Los Baños, Laguna, Philippines (LB, RYT plot), and (2) the Multi-Environment Trial (MET) data set, consisting of 2 years of data (2011–2012), two seasons per year (dry and wet) taken at 7 sites in SE Asia: LB, San Meteo, Isabela, Philippines (I), Munoz, Nueva Ecija, Philippines (NE), RTR, Agusan del Norte, Philippines (A), Ubay, Bohol, Philippines (B), Midsayap, Cotabato Philippines (M), Batalagoda, Sri Lanka (SL) and Hai Dong, Vietnam (V). Flowering time (F/FLW), Plant height (P/PH) and grain yield (Y/YLD) were collected for both data sets, for all sites and seasons, except where marked. *h*^2^=narrow-sense heritability. Data sets were highly unbalanced as is common for large multi-year, multi-site empirical breeding trial data sets, for additional details, see methods section.

**Table 2:** Analysis of IRRI multi-environment (MET) program target population of environments (TPE) for SE Asia irrigated rice

*TRAIT*	*Site*	*Site Lat.*	*F-ratio*	P*-value*	*Highest mean grouping*	*Mean %Δ from all*	*Δ from all sig?*	*Mean %Δ VP site only*	*Δ from VP only sig?*
FLW	M	7.19	8.28	<0.0001	B,M,SL,A	17.96	no	1415.38	yes
FLW	SL	7.53	6.82	0.0002	B,M,SL,A	NS, Δ=0.05	no	266.00	no
FLW	A	8.95	0.06	0.99	B,M,SL,A,LB	5.53	no	5.10	no
FLW	LB	14.17	2.62	0.0383	NE,I,LB,A	1.08	no	23.81	yes
FLW	I	14.70	1.56	0.17	NE,I,LB,A	3.51	no	19.90	no
FLW	NE	15.72	0.81	0.5464	NE,I,A == NE,I,LB,A == ALL	0.00	no	19.03	no
PH	M	7.19	20.88	<0.0001	ALL	0.00	no	136.90	yes
PH	SL	7.53	37.90	<0.0001	B,M,SL,A,LB	7.50	no	6042.86	yes
PH	A	8.95	23.00	<0.0001	NE,I,LB,A == B,M,SL,A	9.24	yes	32.43	yes
PH	B	9.99	6.59	<0.0001	B,M,SL,A	8.22	yes	20.96	yes
PH	LB	14.17	92.97	<0.0001	NE,I,LB,A	3.00	no	100.00	yes
PH	I	14.70	3.14	0.0089	NE,I,LB,A	0.17	no	102.00	yes
PH	NE	15.72	72.20	<0.0001	NE,I,LB,A	0.03	no	2.87	yes
YLD	M	7.19	23.94	<0.0001	B,M,SL,A	2.05	yes	NS, Δ=0.49	yes
YLD	SL	7.53	21.18	<0.0001	B,M,SL,A	78.60	yes	NS, Δ=0.31	yes
YLD	A	8.95	8.81	<0.0001	B,M,SL,A	943.48	yes	98.35	yes
YLD	B	9.99	4.55	0.0007	B,M,SL,A	NS, Δ=0.16	yes	67.39	yes
YLD	LB	14.17	13.59	<0.0001	NE,I,LB,A	50.00	yes	360.00	yes
YLD	I	14.70	5.84	0.0002	NE,I,LB,A	35.48	no	125.00	yes
YLD	NE	15.72	3.46	0.0054	VP site only == NE,I,LB,A	45.05	yes	0.00	no

ANOVA and pairwise Students *t*-test were performed using the CV MET results to model the effect of the combination of sites in the training population on prediction accuracy, by validation site, across all experiments for each trait (Methods section). FLW=flowering time, PH=plant height, YLD=grain yield. Sites= IRRI, Los Baños, Laguna, Philippines (LB), San Meteo, Isabela, Philippines (I), Munoz, Nueva Ecija, Philippines (NE), RTR, Agusan del Norte, Philippines (A), Ubay, Bohol, Philippines (B), Midsayap, Cotabato, Philippines (M), and Batalagoda, Sri Lanka (SL). Site Lat.=latitude of site. F-ratio and *P*-value=F-ratio and *P*-value for ANOVA for each site and trait, respectively. Highest mean grouping=set of sites in training population that produced the highest mean accuracy for a given trait and site across statistical methods and experiments. From this analysis two site groupings emerged, a northern grouping consisting of Nueva Ecija, Isabela, IRRI, and Agusan, and a southern grouping consisting of Agusan, Bohol, Midsayap, and Sri Lanka. Note that whenever the highest mean grouping differed from one of the above two groups, the difference was generally very small and not significant. Mean %Δ from all=% difference in mean accuracy of the best grouping and the mean accuracy of using all sites in the training population. Mean %Δ VP site only=% difference in mean accuracy of the best grouping and the mean accuracy of using only the validation site (VP). NS=no solution due to need to divide by zero, 'Δ' in NS proportions show simple difference between accuracies. A 'yes' in the 'Δ from all sig?' column indicates that the difference in mean accuracy between the best mean grouping and the all sites group was significant, a 'yes' in the 'Δ from VP only sig' column indicates that the difference in mean accuracy between the best mean grouping and using only the VP site in the training population was significant.
