# Supplementary material for: Genome-wide prediction models that incorporate de novo GWAS are a powerful new tool for tropical rice improvement
Source: Heredity (Edinb). 2016 Feb 10;116(4):395–408. doi: 10.1038/hdy.2015.113 (PMC4806696; doi:10.1038/hdy.2015.113)
Supplement: Supplementary Information [file hdy2015113x1.docx]

Genome-wide prediction models that incorporate *de novo* GWAS are a powerful new tool for tropical rice improvement

Supplementary Information

Supplementary note

The authors of Zhang *et al*., 2014 ([Zhang *et al*, 2014](#_ENREF_6)) also made use of the Zhao *et al*., 2011 data ([Zhao *et al*, 2011](#_ENREF_7)), however Zhang *et al* used the Zhao rice diversity panel data as the training and validation population for performing GS cross-validation to test their GS+GWAS models, for which they report prediction accuracies of up to 100%. These fallacious accuracies were obtained because the authors did not control for population structure in this rice diversity panel. Quite the opposite, they performed cross validation *across* the five different subpopulations of rice. Given the extreme differentiation between theses subpopulations, this is a gross mistreatment of the data. Any model trained on such a mixture of subpopulations will predict only the difference between subpopulations, i.e., which subpopulation an individual belongs to, and will thus be both inflated and useless. This was well established empirically using this same dataset by Guo *et al*., 2014 ([Guo *et al*, 2014](#_ENREF_2)).

Supplementary Materials and Methods

**Replicated Yield Trial (RYT) phenotyping**

Phenotypes for the replicated yield trials (RYT) were used for all the experiments and curated from the IRRI database for years 2009-2012, including wet and dry seasons each year. All of the RYT breeding lines, of which our selected 369 lines are a subset, were grown in a randomized complete block design with three replicates in the same field location at IRRI every season and year (Table 1) -- each phenotype was measured for all three replicates. The following data were collected each year, with the exception that plant height data was not available for the 2009 wet season:

- plant height: the actual measurement in cm from soil surface to tip of tallest panicle (awns excluded)
- flowering time: days to when 50% of flowers were visible in whole plot
- maturity date: days to when 85% of grains on panicle were mature
- number of effective tiller or panicle per plant: count of the number of panicles on each plant
- lodging score: percent of plants that lodged
- grain yield (kg/ha): grain yield from a representative plot was harvested and weighed, from this sample the grain yield per hectare was calculated from an inner harvested area of the plot excluding border rows
- rep number: replication number of observation

The plant height, flowering time, and grain yield phenotypes were selected for prediction using the genomic selection models.

**Multi-Environment Testing (MET) program phenotyping**

In 2011, the 369 lines used in this study were planted in a randomized complete block design at IRRI (Los Baños, Laguna, Philippines), San Meteo (Isabela, Philippines), Munoz (Nueva Ecija, Philippines), and RTR (Agusan del Norte, Philippines). In 2012, 203 of the 363 lines were planted at Ubay (Bohol, Philippines), in addition to the same four locations as in 2011. 53 of the 363 lines were also planted at Midsayap (Cotabato Philippines), Batalagoda (Sri Lanka), and Hai Dong (Vietnam) in the 2012 wet season (Table 1).

In the 2011 dry season, the crops at IRRI and Agusan were lost due to pests and flooding, respectively, while in the 2011 wet season, the crops at all sites except Agusan were lost as a result of typhoons across the Philippines. No crops were lost in 2012. See Table 1 for a summary of the missing data. The following phenotype data were collected each year, with the exception that flowering time data was not available for Bohol in the 2012 wet season:

- flowering time: days to when 50% of flowers were visible in whole plot
- maturity date: days to when 85% of grains on panicle were mature
- number of effective tiller or panicle per plant: count of the number of panicles on each plant
- lodging score: percent of plants that lodged
- grain yield (kg/ha): grain yield from a representative plot was harvested and weighed, from this sample the grain yield per hectare was calculated from an inner harvested area of the plot excluding border rows
- rep: replication number of observation
- Row: row number of plot in which plant was grown
- Column: column number of plot in which plant was grown
- PHP: phenotypic acceptability score for the whole plant (not available for Sri Lanka or Vietnam)
- PHPN: phenotypic acceptability score for panicle (available only for 2012WS except at Sri Lanka, Vietnam, and Midsayap)
- PHG: phenotypic acceptability score for grain (available only for 2012WS except at Sri Lanka, Vietnam, and Midsayap)

For additional details on phenotyping see Begum *et al*., 2015.

**GBS data analysis**

SNPs were discovered and called from the raw GBS data using the TASSEL3.0 GBS pipeline with physical alignment to the MSU version 6.0 Nipponbare rice reference genome using Bowtie2, and imputed using TASSEL, as described in Spindel et al., 2015 ([Spindel *et al*, 2015](#_ENREF_5)). Post-imputation, three different datasets were obtained using different filtering parameters. The first dataset, which was used to perform all GS CV experiments in Spindel *et al*., 2015 and calculate the trait heritabilities in Table 1, was obtained by removing all SNPs with call rates < 90%, along with all monomorphic SNPs, for a total SNP set of 73,147 SNPs.

The genotype dataset used for the CV experiments with results reported in Tables S2 and S4 in this publication were obtained by removing all SNPs with call rates < 75% and monomorphic SNPs, for a total SNP set of 108,005 SNPs. The results reported in supplementary tables S2A and S3B are comparable to those obtained using the 73,147 more stringently filtered SNP set used in Spindel *et al*., 2015. We present the results obtained using the 108,005 SNPs here, however, to have a more direct comparison between the RR-BLUP+fixed effects models and those models tested previously. The larger set of SNPs was used for the RR-BLUP+fixed effects models in order maximize our chances of finding the best SNPs to fit as fixed effects in the RR-BLUP+fixed effects models, as based on the GWAS using the individuals in the training population (see "genomic selection modeling" below).

A third dataset, used to run GEMMA for each training fold for the GS + de novo GWAS experiments was obtained by removing all SNPs with call rates < 75%, and SNPs with minor allele frequencies (MAF) < 0.05, for a total of ~58,318 SNPs. This dataset was also used to select SNP subsets for the cross-validation using SNP subsets experiments reported in Table S3.

For all datasets, after SNP filtering, individuals with more than 60% missing data were dropped from the dataset, which resulted in the removal of six individuals that failed sequencing for a total of 363 genotyped lines.

All genotype datasets were transformed from nucleotide genotype coding (i.e., 'A', 'C', 'T', 'G') to numeric coding (1, 0, -1 for class I homozygotes, heterozygotes, and class II homozygotes, respectively) in order to facilitate statistical analysis. The minimal remaining missing data were filled using the numeric genotype means of each line in order to perform PCA and GWAS.

**Cross-validation experimental design (MET data)**

For the MET dataset, each experiment was varied in terms of sites, years, and seasons in the training population, and the site, year, and season of the validation population. The complete listing of all MET experiments is shown in Table S1B. The rows of the table give the combination of sites in the training population, while the columns of the table give the validation season (either 2012 dry season or the 2012 wet season) and the years and seasons in the training population. For experiments that included the 2012 dry season, experiments were tested both with and without the 2012 dry season data for Isabela (when that site was included in the training group, i.e, the row.) Vietnam was excluded from most experiments because data were available on only a few lines, and the results were badly correlated with all other sites (Fig S2).

Due to the unbalanced nature of the dataset, some combinations of rows and columns produced redundant experiments -- these were not performed and are represented on the table as a blank cell. The rest of the cells contain the validation sites run for each experiment, e.g, for experiment 1A, four validation sites were run: Agusan, Bohol, IRRI/Los Baños, and Isabela. All logical combinations of sites, years, and seasons in the training population were tested, see Table S1B.

**Calculation of adjusted phenotypes for validation folds and correlation analyses**

For both the RYT and MET datasets, multiple years, seasons, and replicate yield entries existed along with the previously described covariates for all 332 individuals. In order to build genomic selection models, it was necessary to convert these raw yields into a single, adjusted yield for each individual.

For the correlation analyses and the validation populations, adjusted yields (Kg/HA) were calculated for each Year x Season or each Year x Season x Site combination for the RYT and MET sets, respectively, by fitting an initial linear model of yield (Kg/HA) *y,* by line ID (GHID) *x_1_*_,_ and phenotype covariates described in the above section on phenotyping, *x_2. . .n_* for the given Year x Season or Year x Season x Site combination in JMP v. 10.0. Non-significant covariates as determined by an F-test (α >= 0.05) or covariates that resulted in singularities were removed, and the model re-fit. When all covariates included in the model tested significant, the least squares mean (a.k.a adjusted means/population marginal means) yield for each line ID was exported as the adjusted yield. Adjusted means, in this case, are the phenotype values predicted by the model for each line ID where the other model factors were set to neutral values. For the continuous effects (e.g. flowering time), the neutral value was defined as its sample mean. For the categorical effects (e.g. season), neutral value was defined as the average of the coefficients for that effect. In cases where effect data are missing, the LS means produce more accurate average phenotypes than simple mean. Heat maps of the MET pairwise correlations were produced using JMP (Fig. S2)

**Genomic Selection Modeling**

All statistical modeling was done in R. RR-BLUP models were calculated using package rrBLUP (function kinship.BLUP) ([Endelman, 2011](#_ENREF_1)). RKHS models were calculated using kinship.BLUP, K.method = "GAUSS", modified so that parameter theta was always equal to 2.5, as per guidelines in the BGLR package documentation ([Pérez and de los Campos, 2013](#_ENREF_4)). Random Forest was performed using package randomForest (function randomForest). Bayesian LASSO was performed using package BLR (function BLR).

**Inclusion of validation population year/season in training population**

Cross validation experiment 1 (CV1) accuracies were calculated for all experiments with the validation year/season included in the training population, excluding individuals in the validation fold. Including the validation year/season in the training population can bias accuracies upwards by confounding GxE and line effects, however, so in order to obtain an estimate of this bias, we also performed cross validation experiments 2 and 3 (CV2, CV3) for the RYT experiments. For CV2, we excluded the validation year/season from the training population. These results are not directly comparable to those in which the training population contained the validation year/season (CV1), however, because the training population for CV2 is smaller than was used for CV1 and training population size can have an important effect on prediction accuracy. For this reason, we performed CV3, in which we included the validation year/season in the training population, but removed the equivalent seasons from 2011, e.g., for experiment 1 shown in Table S1A, CV2 would not include the 2012 dry season in the training population, and CV3 *would* include the 2012 dry season but would *not* include the 2011 dry season. Thus, the estimate of bias can be calculated for a given CV permutation experiment as CV3 accuracy minus the CV2 accuracy ([Ly *et al*, 2013](#_ENREF_3)).

As all biases in the RYT dataset were found to be either very small or negative, we did not perform CV2 or CV3 and calculate bias for the MET experiments or CV experiments using marker subsets.

**ANOVA of cross-validation results**

For the RYT dataset, the cross-validation results for each trait were analyzed independently using ANOVA and pairwise student's t to determine:

a. Significant difference in the performance of the different statistical methods across the different experiments by validation season, i.e., where *y_i_* (accuracy) = *μ* + *x_ij_β_j_* + *ε_ij_*, and *i* is one RYT experiment for stat method *j* (e.g. *x_i_* = CV experiment 1 and *j* = RR-BLUP ). Results are shown in the 'GWAS_used' column of Table S2B and over the bars in Figs 1-2., where rows/bars not connected by the same letter within the same trait and validation season indicate significant difference in statistical method performance. Absence of superscript letters indicates that there were no significant differences.

b. Significant difference in performance of training population composition across different statistical methods (excluding the two worst performing methods, MLR and BL) by trait and validation population, i.e., where *y_i_* (accuracy) = *μ* + *x_ij_β_j_* + *ε_ij_*, and *i* is one stat method for training population composition *j* (e.g. *x_i_* = RR-BLUP and *j* = EXP 1). Results are shown in the 'EXP' column of Table S2B, where rows not connected by the same letter within the same trait and validation season indicate significant difference in training population composition performance. Absence of superscript letters indicates that there were no significant differences. The results were largely consistent with those found in Spindel et al., 2015, and thus are not discussed further here.

For the MET dataset, ANOVA and pairwise student's t were used independently for each trait to determine:

a. Significant difference in the performance of the different statistical methods across all sites and experiments by validation season, i.e., where *y_i_* (accuracy) = *μ* + *x_ijk_β_jk_* + *ε_ikj_*, and *i* is one MET experiment for stat method *j* and validation season *k* (e.g. *x_i_* = experiment 1A Agusan, for *j* = RR-BLUP and *k* = 2012 DS ). Results are shown in the 'GWAS_used' columns of Tables S4A-S4B and under the bars of Fig. 4, where rows/bars within the same validation season and trait not connected by the same letter indicate significant difference in statistical method performance. Absence of superscript letters indicates that there were no significant differences.

b. Effect on model performance of the composition of years and seasons in the training population (i.e., Table S1B column) across all sites and experiments by validation season, i.e., where *y_i_* (accuracy) = *μ* + *x_ijk_β_jk_* + *ε_ijk_*, and *i* is one MET experiment for training population year/season composition *j* and validation season *k* (e.g. *x_i_* = experiment 1 Agusan RR-BLUP, for *j* = 2011-2012 all seasons/B and *k*= 2012 DS). Results are shown in the 'Table_S1B_col' columns of Tables S4A and S4B, where capital letters not connected by the same superscript letter indicate a significant difference in the performance of the season/year combination within a given trait. Absence of superscript letters indicates that there were no significant differences.

c. Effect of validation site on model performance across all experiments by validation season, i.e., where *y_i_* (accuracy) = *μ* + *x_ijk_β_jk_* + *ε_ijk_*, and *i* is one MET experiment for validation site *j* and validation season *k* (e.g. *x_i_* = experiment 1A RR-BLUP for *j* = Agusan and *k*= 2012 DS). Results are shown in the 'VP_site' columns of Tables S4A-S4B, where sites within the same validation season and trait not connected by the same superscript letter indicate a significant difference in the performance.

d. Effect of training group (the combination of validation sites in the training population/Table S1B row) on model performance by validation site across all experiments and statistical methods, i.e., where *y_i_* (accuracy) = *μ* + *x_ij_β_jk_* + *ε_ijk_*, and *i* is one MET experiment for Training group *j* and validation site *k* (e.g. *x_i_* = experiment 1A RR-BLUP for *j =* all sites except Vietnam/1 and *k*= Agusan. Results are shown in the ' Table_S1B_row' columns of Tables S4A-S4B, where experiments *belonging to the same validation site* (for the same trait) not connected by the same superscript letter indicate a significant difference in performance of that training group for that site. Note that unlike for tests a-c, this test was not specific to validation season.

**Selection of Random and Distributed SNP subsets**

To select subsets of SNPs that were evenly distributed across the genome, 11 bin parameters were selected: 25Kb (0.1 cM), 50 Kb (0.2 cM), 120 Kb (.5 cM), 240Kb (1 cM), 480 Kb (2 cM), 840 Kb (3.5 cM), 1200 Kb (5 cM), 1800 Kb (7.5 cM), 2400 Kb (10 cM), 3600 Kb (15 cM), 4800 Kb (20 cM). For each bin parameter, all SNPs in the 58,318 SNP set were placed into bins according to the bin parameter. To select subsets of SNPs for a given bin size, the SNPs in each bin were sorted first by minor allele frequency, largest to smallest, and then by call rate, largest to smallest. Ten selections of SNPs were made for each bin size -- the first subset consisted of the top ranked SNP in each bin, i.e., the SNP with the highest MAF and call rate, the second subset consisted of the second ranked SNP in each bin, and so on for the top ten SNPs in each bin. If a bin had fewer than ten SNPs, then the top SNP in each bin was chosen for all ten selections.

To select subsets of SNPs at random, 10 random selections from the 58,318 SNP set for 15 subset sizes: 24, 48, 65, 83, 96, 109, 160, 212, 314, 448, 770, 1492, 2773, 5814, 9729 using a pseudo-random numbers generator. 83, 96, 109, 160, 212, 314, 448, 770, 1492, 2773, 5814, 9729 subsets were chosen to match the number of SNPs in the distributed SNP subsets described above. The additional SNP subset sizes were included to improve resolution.

**Supplementary Figure Legends**

**Figure S1.** Cell plots showing the correlations of the phenotype least square means for sites, seasons, and years in the MET dataset: flowering time (FLW top) plant height (PH, middle), and Grain Yield (YLD, bottom).

**Figure S2.** Heat maps showing pairwise linkage disequilibrium by chromosome.

**Supplementary Table Legends**

**Table S1. (Excel doc)** (A - sheet 1). Description of RYT cross-validation experiments. TP = training population, DS = dry season, WS = wet season, VP = validation population, number in cell = experiment code number. (B - sheet 2). Description of MET cross-validation experiments. Table row gives the training group (combination of sites and seasons in the training population), table column gives the validation season (VP) and combination of years and seasons in the training population. Cells contain the validation sites run (separately) for each experiment. Some cells are blank because the experiments were redundant due to missing data. IRRI/Los Baños = IRRI, Los Baños, Laguna, Philippines, Isabela = San Meteo, Isabela, Philippines, NE = Munoz, Nueva Ecija, Philippines, Agusan = RTR, Agusan del Norte, Philippines, Bohol = Ubay, Bohol, Philippines, Mid = Midsayap**,** Cotabato Philippines, SL = Batalagoda, Sri Lanka), Vietnam = Hai Dong, Vietnam.

**Table S2.** (A, sheet 1). RYT Cross-validation results for prediction of flowering time (FLW), plant height (PH), and grain yield (YLD) using GS + de novo GWAS models and GS + historical GWAS models, as compared to RF and simple RR-BLUP. Table shows results for the best performing CV experiments for each validation season (2012 dry and wet seasons, DS and WS, respectively). TP = training population, all = both seasons. VP = validation season, fixed_SNPs_1 = markers fit as fixed effects for each fold for the GS+GWAS models, GWAS_used = the GWAS dataset used to select the SNPs fit as fixed effects: 2012 DS = GWAS run using the RYT 2012 DS data on training population individuals, 2012 WS = GWAS was run using 2012 WS data on training population individuals, 44K all = previously published GWAS data were used from Zhao *et al*., 2011, the 'all subpopulations' results, 44K indica = the *indica*  subpopulation results from Zhao *et al*. 2011 were used, 44K TRJ = the *tropical japonica* results from Zhao *et al*. 2011 were used. Accuracy_1 = correlation of the predicted GEBV and the phenotype in the validation population, where the training population included the validation season/year for individuals not in the validation fold (CV1)(methods). ave corrected p-val = average p-value of SNPs fit as fixed effects in the GS + de novo GWAS models, after FDR multiple test correction (B, sheet 2). Complete RYT GS cross-validation results for prediction of FLW, PH, and YLD in the 2012 DS and 2012 WS using the GBS dataset consisting of all SNPs post-imputation with call rates >= 75%, = 108,005 SNPs. Accuracy_1 = correlation of the predicted GEBV and the phenotype in the validation population, where the training population included the validation season/year for individuals not in the validation fold (CV1), Accuracy_2 = correlation of the predicted GEBV and the phenotype in the validation population, where the validation year/season is not included in the training population (CV2), Accuracy_3 = correlation of the predicted GEBV and the phenotype in the validation population, where the validation year/season is included in the training population, but the equivalent set of data is removed from year 2011 (CV3) (methods). Bias estimate = Accuracy_3 -Accuracy_2. fixed_snps_1 = markers fit as fixed effects for each fold for the GS + GWAS models for CV1, fixed_snps_2 = markers fit as fixed effects for each fold for the GS + GWAS models for CV2, fixed_snps_3 = markers fit as fixed effects for each fold for the GS + GWAS models for CV3. Other columns are as in (A). 'GWAS_used' rows not connected by the same letter indicate statistical difference in performance of the methods across experiments by validation population, using pairwise students t (α = .05), 'EXP' rows not connected by the same letter indicate statistical difference in performance of the training population compositions/experiments across statistical methods (excluding BL and MLR) by validation population, using pairwise students t (α = .05).

**Table S3. (Excel doc)** CV results for prediction of flowering time (FLW), plant height (PH), and grain yield (YLD) in the 2012 dry season (2012 DS) and the 2012 wet season (2012 WS) using random and distributed SNP subsets. TP = training population from previously best performing experiment, i.e., the training population from Table S2A, VP = validation population, fixed_SNPs= SNPs fit as fixed effects for each fold for the GS + *de novo* GWAS models, set = selection number (out of 10) of either random or distributed SNPs, SNP_num = number of SNPs, bin _size = spacing of distributed SNPs (methods), average corrected p-value = average p-value of SNPs fit as fixed effects in the GS + *de novo* GWAS models, after FDR multiple test correction. SNP numbers not connected by the same letter indicate significant difference in mean prediction accuracy by trait, validation season, and statistical method using pairwise students t (α=0.05).

**Table S4. (Excel doc)** (A sheet 1) Best multi-environment (MET) cross-validation GS results for flowering time (FLW), plant height (PH), and grain yield (YLD). Table gives the results of all statistical method for the best performing experiment (best of which is highlighted in light orange), and the best performing experiment + best statistical method where the training group matched the northern and southern site groupings defined in Table 2 for each validation site and season (highlighted in green). Yellow highlighting indicates that best overall experiment also had a training population consisting of the northern or southern site grouping. ' Table_S1B_row' = numeric code for combination of sites in the training population given in Table S1B, ' Table_S1B_col' = letter code for combination of years and seasons in the training population given in Table S1B. 'VP_site' = site at which validation population data was collected: IRRI= Los Baños = LB, NE= Nueva Ecija, Agusan = Ag, Isabela = Isa, Midsayap = Mid, SL = Sri Lanka, Bohol = Bh, Vietnam = V. 'Training group' = sites included in training population, 'Training pop' = years and seasons included in the training population data, ' SitesxSeasons_IN_training' = listing of the site x year x season data included in the training population, i.e., composite of information in columns B, C, and E, adjusted for missing data. 'Val pop' = validation population, either the 2012 dry season (2012 DS) or the 2012 wet season (2012 WS), Method = statistical method used, GWAS_used= the GWAS dataset used to select the SNPs fit as fixed effects: VP_data = GWAS run using the MET validation site data on the training population individuals, RYT 2012 DS = GWAS run using the RYT 2012 DS data on training population individuals, RYT 2012 WS = GWAS was run using 2012 WS data on training population individuals, 44K_all = previously published GWAS data were used from Zhao et al., 2011, the 'all subpopulations' results, 44K_indica = the *indica*  subpopulation results from Zhao *et al*. 2011 were used, 44K_TRJ = the *tropical japonica* results from Zhao *et al*. 2011 were used. 'Accuracy' = correlation of the predicted GEBV and the phenotype in the validation population, where the training population included the validation season/year for individuals not in the validation fold. 'Fixed SNPs' = SNPs included in the GS + GWAS models as fixed effects for each fold. average_p_wald_corrected = average p-value of SNPs included as fixed effects in GS + de novo GWAS models after FDR multiple test correction. 'Table_S1B_row' cells not connected by the same letter indicate significant difference in effect of training groups on prediction accuracy for a given trait, validation site, and validation season across all experiments, 'Table S1B col' cells not connected by the same letter indicate significant difference effect of training seasons/years on prediction accuracy for a for a given trait, validation site, and validation season across all experiments, 'GWAS_used' cells not connected by the same letter indicate significant difference in performance of statistical methods for a given trait, validation site, and validation season across all experiments, and 'VP site' cells not connected by the same letter indicate significant difference in performance of validation sites for a given trait and validation season across all experiments. (B, sheet 2). All MET CV results for FLW, YLD, and PH with the best performing experiment for each site and validation season highlighted. Columns are as described in A.

Scripts.zip is an file archive containing copies of the R-scripts used to run the various GS models in this publication.

References (Supplement only)

Endelman JB (2011). Ridge regression and other kernels for genomic selection with R package rrBLUP. *Plant Genome* **4:** 250-255.

Guo Z, Tucker D, Basten C, Gandhi H, Ersoz E, Guo B *et al* (2014). The impact of population structure on genomic prediction in stratified populations. *Theor Appl Genet* **127**(3)**:** 749-762.

Ly D, Hamblin M, Rabbi I, Melaku G, Bakare M, Gauch HG *et al* (2013). Relatedness and Genotype × Environment Interaction Affect Prediction Accuracies in Genomic Selection: A Study in Cassava. *Crop Science* **53**(4)**:** 1312.

Pérez P, de los Campos G. (2013). CRAN.

Spindel J, Begum H, Akdemir D, Virk P, Collard B, Redo√±a E *et al* (2015). Genomic Selection and Association Mapping in Rice (<italic>Oryza sativa</italic>): Effect of Trait Genetic Architecture, Training Population Composition, Marker Number and Statistical Model on Accuracy of Rice Genomic Selection in Elite, Tropical Rice Breeding Lines. *PLoS Genet* **11**(2)**:** e1004982.

Zhang Z, Ober U, Erbe M, Zhang H, Gao N, He JL *et al* (2014). Improving the Accuracy of Whole Genome Prediction for Complex Traits Using the Results of Genome Wide Association Studies. *PLoS One* **9**(3).

Zhao K, Tung C-W, Eizenga GC, Wright MH, Ali ML, Price AH *et al* (2011). Genome-wide association mapping reveals a rich genetic architecture of complex traits in Oryza sativa. *Nat Commun* **2:** 467.
