# Supplementary figures and images for: Genome-wide prediction models that incorporate de novo GWAS are a powerful new tool for tropical rice improvement
Source: Heredity (Edinb). 2016 Feb 10;116(4):395–408. doi: 10.1038/hdy.2015.113 (PMC4806696; doi:10.1038/hdy.2015.113)

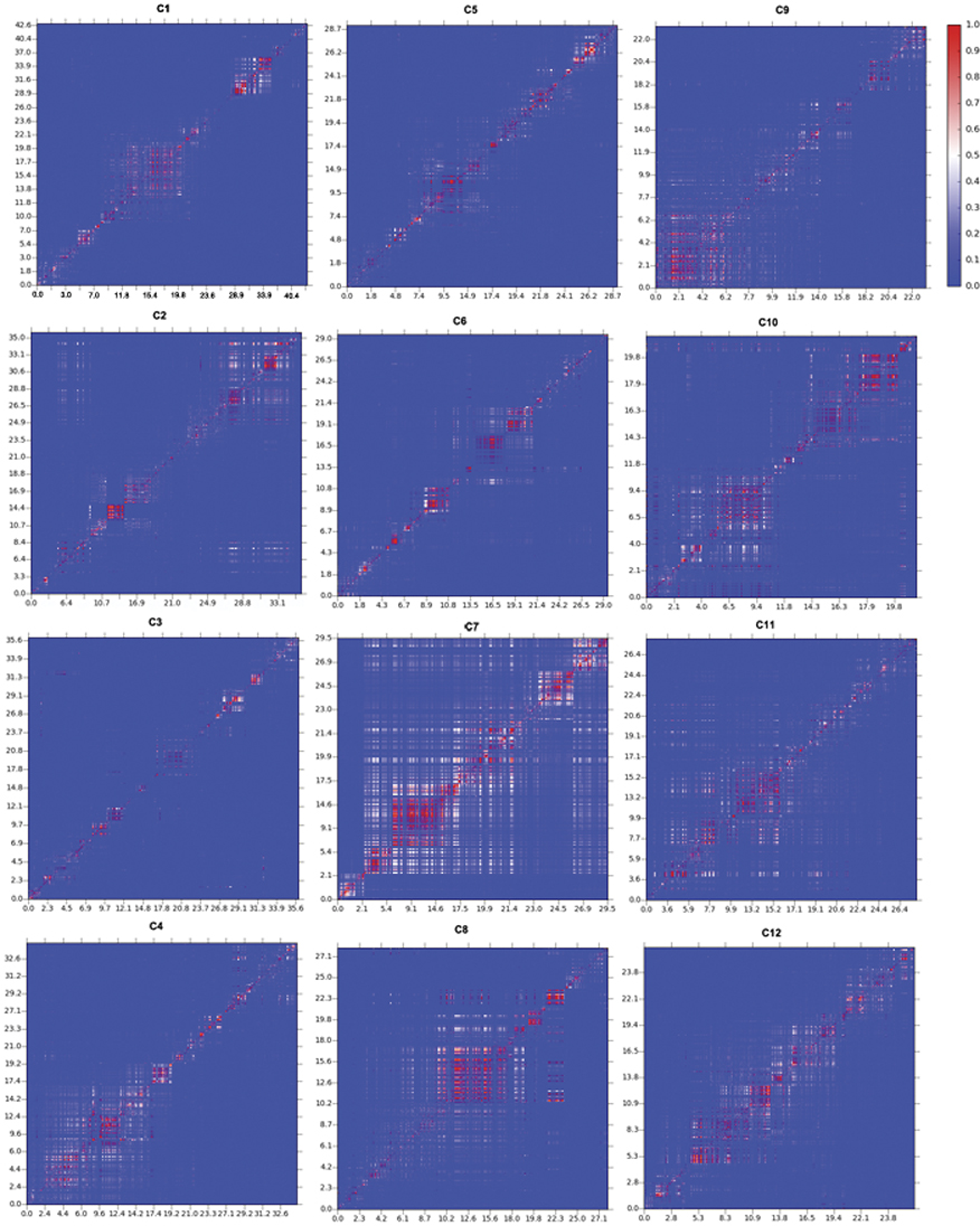

Supplement: Supplementary Figure 2 [file hdy2015113x3.tif]
